# Phylogenomics and morphological evolution of the mega-diverse genus *Artemisia* (Asteraceae: Anthemideae): implications for its circumscription and infrageneric taxonomy

**DOI:** 10.1093/aob/mcad051

**Published:** 2023-03-28

**Authors:** Bohan Jiao, Chen Chen, Meng Wei, Guohao Niu, Jiye Zheng, Guojin Zhang, Jiahao Shen, Daniel Vitales, Joan Vallès, Filip Verloove, Andrey S Erst, Akiko Soejima, Iraj Mehregan, Goro Kokubugata, Gyu-Young Chung, Xuejun Ge, Lianming Gao, Yuan Yuan, Cyprien Joly, Florian Jabbour, Wei Wang, Leila M Shultz, Tiangang Gao

**Affiliations:** State Key Laboratory of Systematic and Evolutionary Botany, Institute of Botany, Chinese Academy of Sciences, Beijing 100093, China; China National Botanical Garden, Beijing 100093, China; University of Chinese Academy of Sciences, Beijing 100049, China; State Key Laboratory of Systematic and Evolutionary Botany, Institute of Botany, Chinese Academy of Sciences, Beijing 100093, China; China National Botanical Garden, Beijing 100093, China; University of Chinese Academy of Sciences, Beijing 100049, China; State Key Laboratory of Systematic and Evolutionary Botany, Institute of Botany, Chinese Academy of Sciences, Beijing 100093, China; China National Botanical Garden, Beijing 100093, China; University of Chinese Academy of Sciences, Beijing 100049, China; State Key Laboratory of Systematic and Evolutionary Botany, Institute of Botany, Chinese Academy of Sciences, Beijing 100093, China; China National Botanical Garden, Beijing 100093, China; University of Chinese Academy of Sciences, Beijing 100049, China; State Key Laboratory of Systematic and Evolutionary Botany, Institute of Botany, Chinese Academy of Sciences, Beijing 100093, China; China National Botanical Garden, Beijing 100093, China; University of Chinese Academy of Sciences, Beijing 100049, China; State Key Laboratory of Systematic and Evolutionary Botany, Institute of Botany, Chinese Academy of Sciences, Beijing 100093, China; China National Botanical Garden, Beijing 100093, China; University of Chinese Academy of Sciences, Beijing 100049, China; State Key Laboratory of Systematic and Evolutionary Botany, Institute of Botany, Chinese Academy of Sciences, Beijing 100093, China; China National Botanical Garden, Beijing 100093, China; University of Chinese Academy of Sciences, Beijing 100049, China; Institute of Botany, Jiangsu Province and Chinese Academy of Sciences (Nanjing Botanical Garden Mem. Sun Yat-Sen), Nanjing 210014, China; Institut Botànic de Barcelona (IBB, CSIC-Ajuntament de Barcelona), Pg. del Migdia, s.n., 08038 Barcelona, Spain; Laboratori de Botànica – Unitat associada al CSIC, Facultat de Farmàcia i Ciències de l'Alimentació -Institut de Recerca de la Biodiversitat (IRBio), Universitat de Barcelona, Av. Joan XXIII 27-31, 08028 Barcelona, Catalonia, Spain; Meise Botanic Garden, Nieuwelaan 38, B-1860 Meise, Belgium; Laboratory Herbarium (NS), Central Siberian Botanical Garden, Russian Academy of Sciences Russia, Novosibirsk, 630090, Zolotodolinskaya st. 101, Russia; Tomsk State University, Laboratoryof Systematics and Phylogeny of Plants (TK), Tomsk 634050, Russia; Faculty of Advanced Science and Technology, Kumamoto University, 2-39-1 Kurokami, Chuo-ku, Kumamoto, 860-8555, Japan; Laboratory for Plant Molecular Phylogeny and Systematics, Department of Biology, Science and Research Branch, Azad University, Tehran, Iran; Department of Botany, National Museum of Nature and Science, Amakubo 4-1-1, Tsukuba, Ibaraki 305-0005, Japan; Department of Forest Science, Andong National University, 1375 Gyeongdong-ro Andong, Gyeongsangbuk-do, 36729, Republic of Korea; Center of Conservation Biology, Core Botanical Gardens, Chinese Academy of Sciences, Guangzhou 510650, China; CAS Key Laboratory for Plant Diversity and Biogeography of East Asia, Kunming Institute of Botany, Chinese Academy of Sciences, Kunming, Yunnan 650201, China; Lijiang National Forest Biodiversity National Observation and Research Station, Kunming Institute of Botany, Chinese Academy of Sciences, Lijiang, Yunnan 67410, China; National Resource Center for Chinese Meteria Medica, Chinese Academy of Chinese Medical Sciences, 100700, Beijing, China; Institut de Systématique Evolution Biodiversité (ISYEB), Muséum national d’Histoire naturelle, CNRS, Sorbonne Université, EPHE, Université des Antilles, 57 rue Cuvier CP39, 75005 Paris, France; Institut de Systématique Evolution Biodiversité (ISYEB), Muséum national d’Histoire naturelle, CNRS, Sorbonne Université, EPHE, Université des Antilles, 57 rue Cuvier CP39, 75005 Paris, France; State Key Laboratory of Systematic and Evolutionary Botany, Institute of Botany, Chinese Academy of Sciences, Beijing 100093, China; China National Botanical Garden, Beijing 100093, China; University of Chinese Academy of Sciences, Beijing 100049, China; Department of Wildland Resources, Utah State University, Logan, UT 84322-5230, USA; State Key Laboratory of Systematic and Evolutionary Botany, Institute of Botany, Chinese Academy of Sciences, Beijing 100093, China; China National Botanical Garden, Beijing 100093, China; University of Chinese Academy of Sciences, Beijing 100049, China

**Keywords:** *Artemisia*, phylogenomics, taxonomy, morphological evolution, generic delimitation, infrageneric taxonomy, genome skimming

## Abstract

**Background and Aims:**

*Artemisia* is a mega-diverse genus consisting of ~400 species. Despite its medicinal importance and ecological significance, a well-resolved phylogeny for global *Artemisia*, a natural generic delimitation and infrageneric taxonomy remain missing, owing to the obstructions from limited taxon sampling and insufficient information on DNA markers. Its morphological characters, such as capitulum, life form and leaf, show marked variations and are widely used in its infrageneric taxonomy. However, their evolution within *Artemisia* is poorly understood. Here, we aimed to reconstruct a well-resolved phylogeny for global *Artemisia* via a phylogenomic approach, to infer the evolutionary patterns of its key morphological characters and to update its circumscription and infrageneric taxonomy.

**Methods:**

We sampled 228 species (258 samples) of *Artemisia* and its allies from both fresh and herbarium collections, covering all the subgenera and its main geographical areas, and conducted a phylogenomic analysis based on nuclear single nucleotide polymorphisms (SNPs) obtained from genome skimming data. Based on the phylogenetic framework, we inferred the possible evolutionary patterns of six key morphological characters widely used in its previous taxonomy.

**Key Results:**

The genus *Kaschgaria* was revealed to be nested in *Artemisia* with strong support. A well-resolved phylogeny of *Artemisia* consisting of eight highly supported clades was recovered, two of which were identified for the first time. Most of the previously recognized subgenera were not supported as monophyletic. Evolutionary inferences based on the six morphological characters showed that different states of these characters originated independently more than once.

**Conclusions:**

The circumscription of *Artemisia* is enlarged to include the genus *Kaschgaria*. The morphological characters traditionally used for the infrageneric taxonomy of *Artemisia* do not match the new phylogenetic tree. They experienced a more complex evolutionary history than previously thought. We propose a revised infrageneric taxonomy of the newly circumscribed *Artemisia*, with eight recognized subgenera to accommodate the new results.

## INTRODUCTION

Reconstructing a well-resolved phylogeny for a mega-diverse genus containing hundreds of species requires two important issues to be addressed. First, molecular markers with sufficient information are needed, especially for genera that have undergone rapid evolutionary radiation (Bagheri *et al.*, 2017; Ma *et al.*, 2018; Heiden *et al.*, 2019). Second, materials (e.g. silica-dried leaves or fresh tissues) are not always available, especially for the genera distributed worldwide (van Welzen *et al.*, 2009; Craven and Biffin, 2010; Frenzke *et al.*, 2015). These limitations have hindered our understanding of the evolution and taxonomy of these mega-diverse genera, which might be important in the economy (e.g. *Syzygium*, Parnell *et al.*, 2007; *Artemisia*, Vallès *et al.*, 2011; *Solanum*, Gagnon *et al.*, 2022), ecology (e.g. *Carex*, Roalson *et al.*, 2021) or conservation (e.g., *Dendrobium*, Niu *et al.*, 2018; Wang *et al.*, 2018).

An approach based on low-depth genomic data can reconstruct the phylogeny of a mega-diverse group (McKain *et al.*, 2018; Xia *et al.*, 2022). Genome skimming (also known as low-coverage genome shotgun sequencing) (Straub *et al.*, 2012; McKain *et al.*, 2018) was first used widely to assemble genomic regions with high copy numbers, such as the chloroplast genome (McPherson *et al.*, 2013; Male *et al.*, 2014), the mitochondrial genome (Guo *et al.*, 2016; Li *et al.*, 2019) and nuclear ribosomal genes (Steele *et al.*, 2012; Zimmer and Wen, 2015). Both fresh and herbarium material (McKain *et al.*, 2018) can be used in this context. This brings great benefits for extensive taxon sampling. At present, a method for obtaining nuclear single nucleotide polymorphisms (SNPs) from genome skimming data based on reference genomes has been developed (Olofsson *et al.*, 2019) and used successfully in Oleaceae and Poaceae (Olofsson *et al.*, 2019; Bianconi *et al.*, 2020; Dong *et al.*, 2022). This economical, convenient method to deal with numerous samples is a potentially powerful tool to solve the phylogeny of mega-diverse genera with published genomes.

**Fig. 1.**
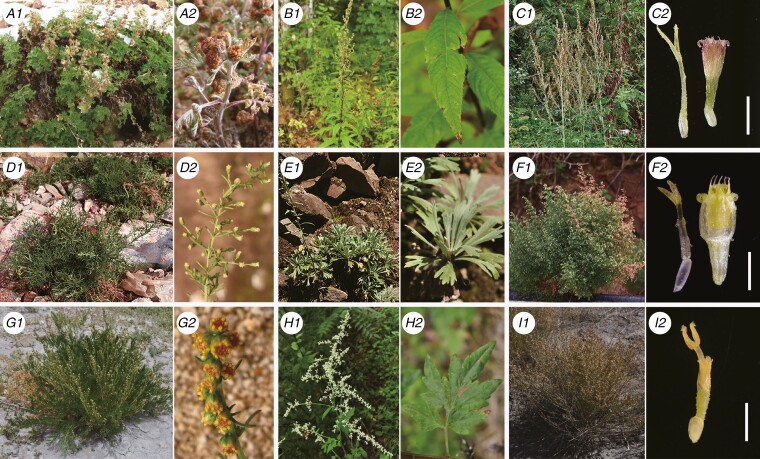
Morphological diversity of nine representative species of *Artemisia*. (A1) *A. gmelinii*; (A2) racemose synflorescence of *A. gmelinii*. (B1) *A. viridissima*; (B2) entire leaf of *A. viridissima*. (C1) *A. chingii*; (C2) a marginal female floret and a disc bisexual floret of *A. chingii*. (D1) *A. fukudo*; (D2) panicle of *A. fukudo*. (E1) *A. lagocephala*; (E2) three-lobed leaf of *A. lagocephala*. (F1) *A. capillaris*; (F2) a marginal female floret and a functionally staminate disc floret of *A. capillaris*. (G1) *A. wellbyi*; (G2) racemose synflorescence of *A. wellbyi*. (H1) *A. lactiflora*; (H2) pinnatisect leaf of *A. lactiflora*. (I1) *A. transiliensis*; (I2) a bisexual floret of *A. transiliensis*. Scale bars: 1 mm.

*Artemisia* (Asteraceae: Anthemideae) is a large genus that has recently undergone rapid evolutionary radiation (Malik *et al.*, 2017). It comprises ~400 species growing in various habitats ranging from desert to wetland, from coasts to rocky beaches, and from arctic to tropical climates (Naithani, 1995; Shultz, 2006; Oberprieler *et al.*, 2009; Ling *et al.*, 2011). It is distributed mainly in the Northern Hemisphere, with a few species extending to South America and Africa (Torrell *et al.*, 1999; Shultz, 2006; Tkach *et al.*, 2008; Ling *et al.*, 2011; Garcia *et al.*, 2011*b*; Malik *et al.*, 2017; Fig. 1). Many *Artemisia* species are economically valuable for their uses in medicine, food, horticulture or ecological restoration. *Artemisia annua* is the most famous species, owing to its antimalarial, artemisinin (Bhakuni *et al.*, 2002; Tu, 2011). The same species is among the plants with evidence suggesting a potential use for the coronavirus disease 2019 pandemic (Nair *et al.*, 2021). *Artemisia anomala*, *A. argyi*, *A. capillaris*, *A. copa* and *A. herba-alba* are traditional medicinal plants (Wright, 2002; Ling *et al.*, 2011; Gras *et al.*, 2020; Mercado *et al.*, 2021). The polysaccharides in the fruits of *Artemisia sphaerocephala* can be used as a food additive (Kakar *et al.*, 2021). *Artemisia dracunculus*, *A. vulgaris*, *A. absinthium* and *A. abrotanum* are widely used for seasoning purposes (Wright, 2002; Shultz, 2006; Ling *et al.*, 2011). Some species, such as *Artemisia ludoviciana* and *A. schmidtiana*, are popular garden plants (Vallès *et al.*, 2011). Some shrubby species, such as *Artemisia ordosica*, are used to stabilize quicksand in deserts (Shultz, 2009; Ling *et al.*, 2011). The enormous value of *Artemisia* species has sparked the deep and continuous interest of researchers from many fields, such as phytochemistry, pharmacology, ecology, agronomy and ethnobotany. A complete and updated taxonomy of *Artemisia* would undoubtedly help us to explore its huge potential value.

The genus *Artemisia* was first described by Linnaeus (1753). It is characterized by having: two types of capitula [heterogamous-disciform capitula (disc florets bisexual or functionally staminate, ray florets pistillate) or homogamous-discoid capitula (disc florets bisexual and fertile, ray florets absent)] (Fig. 1); pollen with short spines or no spines (the so-called *Artemisia* pollen type, Martín *et al.*, 2003); and cypselae without ribs (Linnaeus, 1754, 1767; Bremer and Humphries, 1993; Vallès *et al.*, 2011). However, these characters are not diagnostic for *Artemisia*. Thirteen small or monotypic genera, namely *Artemisiastrum* Rydb., *Artemisiella* Ghafoor, *Crossostephium* Less., *Elachanthemum* Y.Ling & Y.R.Ling, *Filifolium* Kitam., *Hippolytia* Poljakov, *Kaschgaria* Poljakov, *Mausolea* Poljakov, *Neopallasia* Poljakov, *Picrothamnus* Nutt., *Stilpnolepis* Krasch., *Sphaeromeria* Nutt. and *Turaniphytum* Poljakov, were once morphologically related to or merged with *Artemisia* (Poljakov, 1961*a*, 1961*b* and references therein; Heywood and Humphries, 1977; Ghafoor, 1992, 2002; Bremer and Humphries, 1993). To date, the circumscription of the genus *Artemisia* remains controversial and unclear.

Within *Artemisia*, some large infrageneric groups (as sections, subgenera or others) were gradually proposed by different taxonomists at different times (i.e. the four groups *Artemisia*, *Absinthium*, *Dracunculus* and *Seriphidium*). They were accepted or partly revised by later taxonomists based on their research on the *Artemisia* species from a certain region or group (see detailed taxonomy in Table 1, and morphological characters of each infrageneric group in Table 2). In addition to the four groups above, a fifth group, section *Tridentatae* Rydberg, was separated from *Seriphidium* and established by Rydberg (1916). Later on, McArthur *et al.* (1981) raised this group as a subgenus, considering its special geographical distribution (North America), woody life form and unique karyotypic and chemotaxonomic attributes. However, Ling (1982, 1991*a*, 1991*b*) clustered *Seriphidium* and *Tridentatae* as an independent genus, *Seriphidium* (Besser ex Less.) Fourr. Thereafter, the sixth subgenus, subgenus *Pacifica*, was proposed by Hobbs and Baldwin (2013) based on the phylogeny using four molecular markers [two nuclear ribosomal (ITS + ETS) and two chloroplast markers (*trn*L-F + *psb*A–*trn*H)]. It shares the same capitulum type as subgenus *Artemisia* but differs in its ribbed cypselae (vs. not ribbed in subgenus *Artemisia*). It contains only four species distributed in Southeast Asia and on the Hawaiian Islands. In the same publication, Hobbs and Baldwin (2013) recognized six subgenera within *Artemisia* (Tables 1 and 2). In summary, although various classification systems have been proposed for its infrageneric taxonomy (Table 1), a global and generally accepted infrageneric classification system for *Artemisia* based on a robust phylogeny remains missing.

**Table 1.** Comparison of different infrageneric taxonomies of *Artemisia based on**Vallès and McArthur (2001)**and**Riggins (2008)*

Rank	Taxa						Reference
Genus	*Artemisia*						Linnaeus (1753)
Genera	*Artemisia*					*Oligosporus*	Cassini (1817); Lessing (1832)
Sections	*Absinthium*	*Abrotanum*		*Seriphidium*		*Dracunculus*	Besser (1829, 1834, 1835) Candolle (1838); Ledebour (1844–1846)
Sections	*Euartemisia*			*Seriphidium*		*Dracunculus*	Gray (1886)
Subgenera	*Euartemisia*			*Seriphidium*		*Euartemisia*	Rouy (1903)
Subgenera Sections	*Absinthium*	*Abrotanum*		*Seriphidium* *Seriphidium* *Tridentatae*		*Dracunculus*	Rydberg (1916)
Subgenera	*Artemisia*			*Seriphidium*		*Dracunculus*	Poljakov (1961*a*)
Subgenera	*Absinthium*	*Artemisia*		*Seriphidium*		*Dracunculus*	Persson (1974)
Sections	*Artemisia*					*Dracunculus*	Tutin *et al.* (1976)
Subgenera	*Artemisia*			*Seriphidium*	*Tridentatae*	*Dracunculus*	McArthur *et al.*, (1981)
Subgenera	*Artemisia*			*Seriphidium*		*Dracunculus*	Podlech (1986)
Genera Subgenera	*Artemisia* *Artemisia*			*Seriphidium* *Seriphidium*		*Artemisia* *Dracunculus*	Ling (1991*a*, *b*); Ling *et al.* (2011)
Subgenera	*Absinthium*	*Artemisia*		*Seriphidium*	*Tridentatae*	*Dracunculus*	Shultz (2006)
Subgenera	*Absinthium*	*Artemisia*	*Pacifica*	*Seriphidium*	*Tridentatae*	*Dracunculus*	Hobbs and Baldwin (2013)

**Table 2.** Infrageneric taxonomy of *Artemisia*, their morphology and distribution based on Shultz (2006) and Hobbs and Baldwin (2013)

Infrageneric taxa	Morphological characters	Distribution
Subgenus *Artemisia*	Capitulum with outer florets female, central florets bisexual and fertile, receptacle glabrous	Worldwide
Subgenus *Absinthium*	Capitulum with outer florets female, central florets bisexual and fertile, receptacle hairy	Northern Hemisphere
Subgenus *Dracunculus*	Capitulum with outer florets female, central florets bisexual but functionally staminate (not setting fruits), receptacle glabrous	Northern Hemisphere
Subgenus *Seriphidium*	Capitulum without outer florets, florets bisexual and fertile, receptacle glabrous	Eurasia and North Africa
Subgenus *Tridentatae*	Capitulum without outer florets, florets bisexual and fertile, receptacle glabrous	North America
Subgenus *Pacifica*	Capitulum with outer florets female, central florets bisexual and fertile, receptacle glabrous, cypselae ribbed	East Asian Coast and Hawaiian Islands

In recent decades, molecular phylogenetic studies of *Artemisia* have made significant progress and improved our understanding of the phylogeny and taxonomy of *Artemisia*. Among the 13 closely related genera, eight genera (i.e. *Sphaeromeria*, *Artemisiastrum*, *Crossostephium*, *Filifolium*, *Mausolea*, *Neopallasia*, *Picrothamnus* and *Turaniphytum*) were revealed to be nested in *Artemisia* and proposed to be reduced into *Artemisia* (Watson *et al.*, 2002; Sanz *et al.*, 2008, 2011; Garcia *et al.*, 2011*a*, 2011*b*; Pellicer *et al.*, 2011; Sonboli *et al.*, 2012; Hobbs and Baldwin, 2013), whereas three genera (i.e. *Elachanthemum*, *Hippolytia* and *Stilpnolepis*) have a distant relationship with *Artemisia* (Watson *et al.*, 2002; Sanz *et al.*, 2008). However, owing to the limited phylogenetic information of the DNA markers previously used, the relationship among *Artemisia* and the other two genera, *Artemisiella* and *Kaschgaria*, is still controversial; the infrageneric relationships, especially those in the species-rich groups, such as the subgenera *Dracunculus* and *Seriphidium* (Pellicer *et al.*, 2011; Malik *et al.*, 2017), are not well resolved. A well-resolved phylogeny for *Artemisia* with a global sampling remains missing.

Furthermore, molecular systematic studies have revealed increasing conflicts between molecular phylogeny and infrageneric taxonomy of *Artemisia*. With the only exception of subgenus *Pacifica*, the other subgenera of *Artemisia* were not supported as monophyletic (Watson *et al.*, 2002; Vallès *et al.*, 2003; Sanz *et al.*, 2008, 2011; Tkach *et al.*, 2008; Pellicer *et al.*, 2011; Riggins and Seigler, 2012; Hobbs and Baldwin, 2013; Malik *et al.*, 2017). For example, some species of subgenus *Artemisia* were embedded in the subgenus *Absinthium* (figure 1 of the paper by Malik *et al.*, 2017), and some New World species of subgenus *Artemisia* were nested in subgenus *Tridentatae* (Pellicer *et al.*, 2010; Garcia *et al.*, 2011*b*). The infrageneric taxonomy of *Artemisia* including the six subgenera described above is based mainly on morphological characters (Table 2). Traditionally, pollen type and floret functional sex spatial arrangement within the capitula are used to circumscribe the genus *Artemisia* (Bremer and Humphries, 1993; Watson *et al.*, 2002; Vallès *et al.*, 2011). Capitulum type was the main character for its infrageneric taxonomy (Table 2). Other characters, such as life form and leaf shape, although less commonly used than capitulum type, are often used for its subgeneric division (Poljakov, 1961*a*; Tutin *et al.*, 1976; Shultz, 2006, 2009; Ling *et al.*, 2011). For example, life form can be used to define subgenus *Tridentatae*, all species of which are shrubs (Shultz, 2006). Leaf shape is often used for its interspecific taxonomy, and even section taxonomy of subgenus *Artemisia* (e.g. Ling *et al.*, 2011). The conflicts between molecular phylogeny and morphological taxonomy of *Artemisia* made some authors question whether the current infrageneric taxonomy reflected the evolutionary relationships among lineages (e.g. Persson, 1974; Vallès and McArthur, 2001; Shultz, 2006; Garcia *et al.*, 2011*b*) and whether the morphological characters used were reliable (Riggins and Seigler, 2012). Therefore, it is necessary to investigate the evolutionary patterns of these morphological characters in a phylogenetic context and to evaluate their taxonomic value for the generic and infrageneric circumscriptions of *Artemisia*.

In this study, we used a genome-skimming sequencing technique to obtain nuclear SNP data from fresh and herbarium materials of *Artemisia*. Our objectives were as follows: (1) to clarify the circumscription of *Artemisia*; (2) to build a robust phylogeny for *Artemisia* based on a global and representative sampling; (3) to infer evolutionary patterns of six key morphological characters; and (4) to update the infrageneric taxonomy for *Artemisia*. This study will provide a solid foundation for further systematic and evolutionary studies on *Artemisia* and help us to explore its tremendous value.

## MATERIALS AND METHODS

### Taxon sampling

We obtained 205 species of *Artemisia*, representing all six subgenera and covering the distribution area of the genus (Eurasia, North America, Africa and South America). Thirteen samples from the 12 segregated genera (*Artemisiella*, *Crossostephium*, *Elachanthemum*, *Filifolium*, *Hippolytia*, *Kaschgaria*, *Mausolea*, *Neopallasia*, *Picrothamnus*, *Sphaeromeria*, *Stilpnolepis* and *Turaniphytum*; only *Artemisiastrum* missing) were sampled to clarify the circumscription of *Artemisia*. We also sampled ten species from six genera of the subtribe Artemisiinae, including *Ajania* Poljakov, *Allardia* Decne., *Cancrinia* Kar. & Kir., *Chrysanthemum* L., *Nipponanthemum* Kitam. and *Richteria* Kar. & Kir., as outgroups (Watson *et al.*, 2002). All samples were obtained from our field collections or from herbaria (AL, ANH, BC, BCN, BORZ, KUN, PE and UTC; Holmgren *et al.*, 1990). Supplementary data Table S1 provides detailed sampling information.

### Sequencing and nuclear SNP calling

Total genomic DNA was extracted from silica gel-dried leaves or herbarium specimens using the TIANGEN plant genomic DNA extraction kit (TIAN-GEN Biotech., Beijing, China) following the manufacturer’s protocol. Total DNA extracted from silica gel-dried leaves was sheared into ~350 bp fragments to build 350 bp insert libraries, and unsheared DNA from herbarium specimens was used to construct 150 bp insert libraries. The DNA libraries were constructed using the NexteraXT DNA Library Preparation Kit (Illumina, Shanghai, China) and were sequenced on the Illumina HiSeq Xten platform (Illumina, Shanghai, China). We obtained ~3 Gb of data for each sample with paired-end libraries. The average length of the generated reads from silica gel-dried and herbarium specimens was 150 and 100 bp, respectively. The raw sequencing data were checked using FastQC v.0.10.1 (https://www.bioinformatics.babraham.ac.uk/projects/fastqc/).

Nuclear SNPs were obtained using a reference-based approach, following the pipeline of Olofsson *et al.* (2019). Different reference genomes will affect the results of read mapping and SNP calling. To reduce the complexity of the reference genome, we prepared a genome-wide reference data set of putative orthologous sequences using the complete coding sequence (CDS) data sets of *Artemisia annua* (Shen *et al.*, 2018) and *Chrysanthemum seticuspe* (Hirakawa *et al.*, 2019), a species from the closely related genus *Chrysanthemum*. The BLAST reciprocal best hits (RBH) tool (Cock *et al.*, 2015) in BLAST v.2.2.28 (Altschul *et al.*, 1990) was used to select putative one-to-one orthologues (e-value < 1e-10). A total of 22 545 putative one-to-one orthologues were retained. Each of these genes is expected to descend from a single gene in the common ancestor of *Artemisia* and *Chrysanthemum* but might have been lost or duplicated in some derived groups. Collapsing such duplicates allows the extraction of phylogenetically useful markers (Bianconi *et al.*, 2020). Compared with *C. seticuspe*, *A. annua* was more closely related to other *Artemisia* species. We therefore used *A. annua* to conduct downstream analyses.

The first step was to clean and trim raw reads using the NGS QC toolkit v.2.3.3 (Patel and Jain, 2012). Reads with ambiguous base calls and reads with >20 % of the bases having a quality score <20 were removed. Low-quality bases (quality score < 20) were trimmed from the 3ʹ end of each read. Second, the cleaned reads were mapped onto *A. annua* CDS references using BOWTIE2 v.1.1.1 (Langmead and Salzberg, 2012) with the default settings for pair-end reads. The genomic position of each high-quality nuclear SNP was determined using the mpileup function in SAMtools (Li *et al.*, 2009) and the consensus variant caller algorithm in BCFtools v.1.3.1 (Li, 2011). Given that the ploidy levels of the samples were unknown, all samples were treated as diploids, and only SNPs with a maximum of two alleles in the sample were retained. This might lead to the omission of some loci in allopolyploids but does not significantly affect the SNP calling efficiency of the autopolyploids. Treating all samples, even polyploids, as diploids might also increase the frequency of allelic loss in polyploids owing to unequal alignments between different alleles. However, loss of alleles owing to low sequencing depth might be more frequent than loss of loci owing to polyploidy (Olofsson *et al.*, 2016, 2019). Thus, recent studies have shown that treating all samples as diploids has no apparent effect on tree topology in low-coverage data (Olofsson *et al.*, 2016, 2019; Bianconi *et al.*, 2020). For each sample, the median coverage of all SNPs with taxon occurrences >50 % was calculated using a Perl script (supplemental material 2 of the paper by Olofsson *et al.*, 2019). Only loci with coverage between 0.5 and 2 times the median coverage and a minimum quality score of 20 were retained. By controlling the upper threshold of coverage, reads derived from repetitive regions of the nuclear genome or organelle genome can be excluded. Finally, we merged individual genotypes using BCFtools and filtered SNPs that had been shared less than three time using VCFtools v.0.1.14 (Danecek *et al.*, 2011) to exclude erroneous SNP sites caused by low coverage and sequencing errors. Given that phylogenomic analyses can be biased by the reference and the amount of missing data (Bertels *et al.*, 2014; Xi *et al.*, 2016; Olofsson *et al.*, 2019), we repeated the mapping and filtering with different filtering stringencies and an alternative reference species (*C. seticuspe*).

### Phylogenetic analyses

The phylogenetic reconstructions of the nuclear SNP data set were inferred using supermatrix and supertree methods. We used IQtree v.1.6.1 (Nguyen *et al.*, 2015) to build a maximum likelihood (ML) tree. Substitution models were selected based on the corrected Akaike’s information criterion (AICc) calculated in ModelFinder (Kalyaanamoorthy *et al.*, 2017) in IQ-TREE. The supertree method was implemented using ASTRAL III (Mirarab *et al.*, 2014). Only gene alignments ≥150 bp and containing ≥50 % of the total number of samples were used to build single gene trees. We used RAxML v.8.2.4 (Stamatakis, 2014) with a GTR+CAT substitution model and 100 bootstrap pseudoreplicates to infer ML trees for each selected gene alignment. To remove poorly resolved topologies in gene trees, branches with bootstrap support (BS) ≤20 % were collapsed using the ‘nw_ed’ function in Newick Utilities (Junier and Zdobnov, 2010).

### Evolutionary inferences of morphological characters

Here, we investigated these six traits characteristic of different taxonomic ranks of *Artemisia* (Table 3) and reconstructed their ancestral states. The character states of each species were obtained from our observations on living plants and/or herbarium specimens (AL, ANH, BC, BCN, BM, BORZ, BRNU, E, HIB, IBSC, IBK, K, KUN, KYO, P, PE, PR, PRC, TI, TNS and UTC; Holmgren *et al.*, 1990) and the literature (Poljakov, 1961*a*; Tutin *et al.*, 1976; Korobkov, 1987; Krasnoborov, 1997; Shultz, 2006; Ling *et al.*, 2011; Malik and Hayat, 2019). Five of these six characters are discrete, except leaf size. An approximation of leaf size can be obtained by measuring the length and width of the leaf and multiplying the length × width × 3/4 (Cain and Castro, 1959). We transformed this quantitative character into a discrete character according to Webb (1959): a leaf area of <225 mm^2^ was defined as small leaf, ≥225 mm^2^ and <2025 mm^2^ as a medium leaf, and ≥2025 mm^2^ as a large leaf (Table 3).

**Table 3.** Morphological characters and character states of *Artemisia* used in the present study

No.	Character	Character states
1	Pollen type	(A) *Artemisia* type; (B) *Anthemis* type
2	Synflorescence type	(A) Panicle; (B) raceme; (C) corymb
3	Capitulum type	(A) Type 1, heterogamous-disciform; (B) Type 2, heterogamous-disciform, receptacle pubescent; (C) Type 3, heterogamous-disciform with central floret male; (D) Type 4, homogamous-discoid
4	Life form	(A) Annual herb; (B) perennial herb; (C) subshrub/shrub
5	Basal leaf morphology	(A) Entire or three-lobed; (B) pinnatisect, segments < 6 pairs; (C) pinnatisect, segments ≥ 6 pairs
6	Basal leaf size	(A) < 225 mm^2^; (B) 225 mm^2^ ≤ basal leaf size < 2025 mm^2^; (C) basal leaf size ≥ 2025 mm^2^

The ML method was used to reconstruct ancestral states of these six polymorphic characters implemented in RASP v.3.2 (Yu *et al.*, 2015) using the ape package (Paradis and Schliep, 2019) in R (R Core Team, 2019). The character states are provided in Table 3.

## RESULTS

### Nuclear SNP data sets

Using the *A. annua* CDS genome as reference, considering SNPs with <80 % of missing data, we obtained an 615 009 bp SNP alignment, including 258 samples (for each sample, total SNP number range = 79 582–546 927, 95 % range = 379 464–400 124, average = 389 794), including 585 386 parsimony-informative sites. The rates of missing data varied across samples, ranging from 11.07 to 87.06 % (95 % range = 34.89–38.26 %, average = 36.57%). To observe the effect of the amount of missing data per SNP, nine gradients of missing data rates of 10–90 % were set at intervals of ten. No SNPs were retained when a maximum of 10–40 % missing data was allowed. Higher levels of missing data (50–90 %) retained more SNPs (Supplementary data Table S2). Similar observations were made regarding the numbers of SNPs obtained when using the simplified CDS gene of *C. seticuspe* as the reference genome (Supplementary data Table S2).

Using the *A. annua* CDS genome as reference, considering SNPs with <80 % of missing data, we obtained 544 single-gene matrices with a length >150 bp and a species coverage >50 %. In the same conditions and using the *C. seticuspe* CDS genome as reference, 176 single-gene matrices were obtained.

### Phylogenetic relationships

**Fig. 2.**
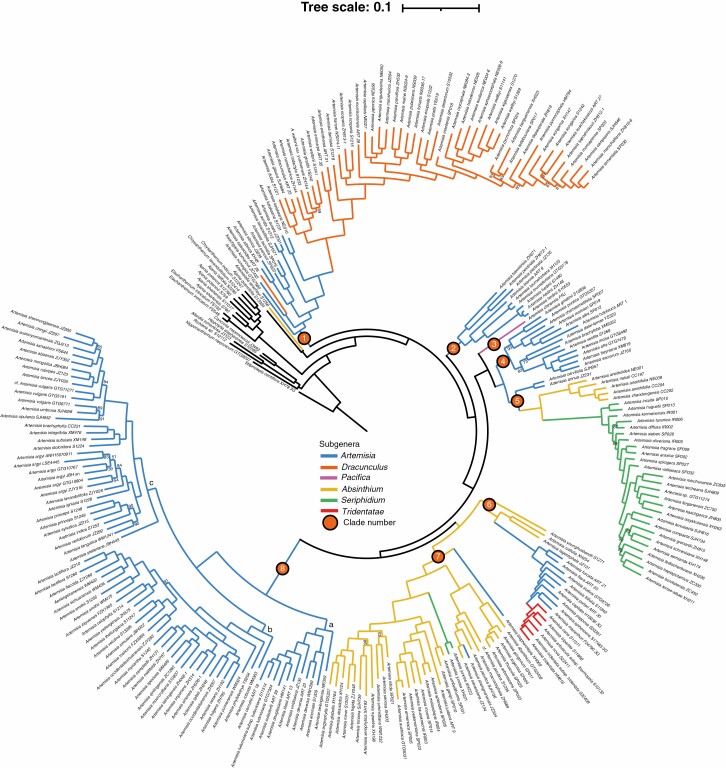
Maximum likelihood phylogenetic tree obtained from the alignment of nuclear single nucleotide polymorphisms (SNPs) obtained by mapping low-depth whole-genome sequencing data reads to the *Artemisia annua* simplified coding sequences (CDS). Node supports were evaluated with 100 bootstrap replicates; bootstrap support (BS) values are indicated along branches (values equal to 100 % are not shown). The colours of branches indicate the traditional subgeneric taxonomy of *Artemisia*. Clades are numbered and denoted by coloured circles.

The topologies inferred from the SNP alignments obtained from different references (*A. annua* and *C. seticuspe* CDS), with different reconstruction methods and including different levels of missing data (50–90 %) were highly similar (Fig. 2; Supplementary data Figs S1–S11). We chose the ML tree inferred from the SNP alignments based on the *A. annua* CDS reference, including 80 % of missing data (Fig. 2) for subsequent discussion, because it had the smallest difference from all other topologies (the smallest Robinson-Foulds distance; Supplementary data Figs S1–S11). Except for the presence of a few tips with relatively low supports (39 % < BS < 95 %; Fig. 2), the topology of this tree was almost totally resolved (BS > 95 %; Fig. 2).

All ML phylogenetic trees showed that the clade consisting of *Ajania quercifolia* (*= Phaeostigma quercifolium*) and *Artemisiella stracheyi* was the sister group of *Artemisia* (Fig. 2; Supplementary data Figs S1–S9), and coalescent species trees showed that these two species were nested in *Artemisia* (Supplementary data Figs S10 and S11; local posterior probability (LPP) = 0.93, 0.65). All analyses showed that *Kaschgaria komarovii* was nested in *Artemisia* and sister to *Artemisia salsoloides* (BS = 100 %; Fig. 2; Supplementary data Figs S1–S9). Coalescent species trees also supported that *K. komarovii* was nested within the *Artemisia* clade, but its exact position within *Artemisia* was unresolved (Supplementary data Figs S10 and S11). The clade consisting of all sampled species of *Artemisia*, *Kaschgaria*, *Ajania quercifolia* and *Artemisiella stracheyi* was the sister to the *Ajania*–*Chrysanthemum*–*Elachanthemum* clade (BS = 100 %; Fig. 2; Supplementary data Figs S1–S4).

All ML analyses revealed that five of the six subgenera of *Artemisia* previously recognized were not supported as monophyletic, with the only exception being the subgenus *Pacifica* (Fig. 2). Among these five subgenera, subgenera *Artemisia* and *Absinthium* are clearly not monophyletic and need to be subdivided greatly, and the other three subgenera, *Dracunculus*, *Seriphidium* and *Tridentatae*, would be monophyletic provided that a few species are removed or added (Fig. 2). In our new analysis, the genus *Artemisia* was split into eight highly supported clades (Fig. 2; BS = 100 %), i.e. Clades 1–8. Among them, Clade 1 and Clade 2 formed the earliest-diverging clades in the genus. Clades 3, 4 and 5 formed a monophyletic group, and together were sister to the monophyletic group consisting of Clades 6, 7 and 8. Clades 4 and 5 were grouped together, and together were sister to Clade 3. Clades 6 and 7 were grouped together, and together were sister to Clade 8 (Fig. 2).

Coalescent analyses also revealed the same eight clades, but some relationships among clades were different or unresolved. The coalescent species trees showed that Clade 1 (LPP = 1, 0.44), Clade 2 (LPP = 0.72, 0.62) and Clade 7 (LPP = 1, 0.55) formed an early-diverging grade in the genus. The monophyletic group consisting of Clades 3, 4 and 5 (LPP = 0.5, 0.63) was sister to the clade consisting of Clades 6 and 8 (LPP = 0.93, 0.65; Supplementary data Figs S10 and S11).

Below, we describe these eight clades of *Artemisia* according to the infrageneric taxonomy including six subgenera (Table 2). All the relationships mentioned in the following results were strongly supported (BS = 100 %); exceptions are highlighted.

**Clade 1** consisted of the entire subgenus *Dracunculus* and some species from subgenus *Artemisia*, plus *K. komarovii*, *Artemisia sibirica* (*= Filifolium sibiricum*), *A. eriocarpa* (*= Mausolea eriocarpa*) and *A. eranthema* (*= Turaniphytum eranthemum*). *Artemisia* subgenus *Dracunculus* was shown to be monophyletic provided that *A. salsoloides* was excluded. The *Dracunculus* clade was sister to *A. keiskeana*, a species of subgenus *Artemisia*.

**Clade 2** included four species of subgenus *Artemisia* (*A. hedinii*, *A. tournefortiana*, *A. biennis* and *A. baxoiensis*) and *A. pectinata* (= *Neopallasia pectinata*). *Artemisia pectinata* was sister to *A. baxoiensis*. This two-species clade was sister to the other three-species clade.

**Clade 3** contained the sampled species of subgenus *Pacifica*, i.e. *A. chinensis* (= *Crossostephium chinense*).

**Clade 4** All species of Clade 4 belonged to subgenus *Artemisia*.

**Clade 5** included nearly all the species of subgenus *Seriphidium* (except *Artemisia juncea*, placed in Clade 7), four species of subgenus *Absinthium* (*A. anethifolia*, *A. anethoides A. zhaodongensis* and *A. nakaii*) and two species of subgenus *Artemisia* (*A. annua* and *A. carvifolia*).

**Clade 6** included the entire North American endemic subgenus *Tridentatae* (*sensu*Garcia *et al.*, 2011*a*, *b*; including *Picrothamnus* and *Sphaeromeria*), three species of subgenus *Artemisia* (*A. flava*, *A. furcata* and *A. sodiroi*) and three species of subgenus *Absinthium* (*A. lagocephala*, *A. rutifolia* and *A. younghusbandii*).

**Clade 7** consisted mainly of species of subgenus *Absinthium*. The first exception was *A. blepharolepis*, a species of subgenus *Artemisia*, which formed the earliest-diverging lineage of Clade 7. The second exception, *Artemisia juncea*, a species of subgenus *Seriphidium*, was also nested in Clade 7.

**Clade 8.** The species of Clade 8 all belonged to subgenus *Artemisia* and could be divided into three subclades (Fig. 2 Clade 8a–c). Clade 8a consisted of some species from East Asia (*A. viridissima*, *A. deversa*, *A. anomala* and *A. selengensis*) and of a subclade comprising all the New World species (*A. tilesii*, *A. douglasiana*, *A. suksdorfii*, *A. ludoviciana* and *A. carruthii*). Clades 8b and 8c were sisters, and together they formed the sister group of Clade 8a. All species of Clades 8b and 8c were distributed in Eurasia.

### Evolution patterns of morphological characters in *Artemisia*

Ancestral state reconstructions were undertaken using the ML tree inferred from the SNP alignments based on the *A. annua* CDS reference, with 80 % missing data (Fig. 2).

#### Pollen type.

**Fig. 3.**
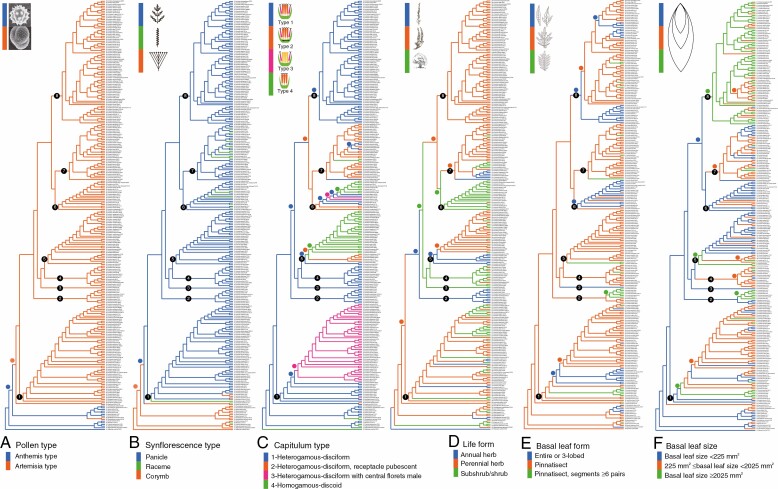
Evolution of six key morphological characters in *Artemisia* and its allies, showing the most likely ancestral character characters implemented in RASP v.3.2 (Yu *et al.*, 2015) using the maximal likelihood method based on the new inferred tree in Fig. 2. (A) Pollen type. (B) Synflorescence type. (C) Capitulum type. (D) Life form. (E) Basal leaf form. (F) Basal leaf size. Detailed probabilities of character states for each node are shown in Supplementary data Figs S12–S17. Colours of dots on the nodes and branches indicate the states.

*Artemisia* pollen type was recovered as the ancestral state of the genus *Artemisia* and is a synapomorphy of the latter clade (Fig. 3A; Supplementary data Fig. S12). *Anthemis* pollen type was the ancestral state for the set of taxa we analysed. The *Artemisia* pollen type originated independently twice from the *Anthemis* pollen type, once in the lineage leading to *Artemisia* and a second time in the ancestor of *Elachanthemum* (Fig. 3A; Supplementary data Fig. S12).

#### Synflorescence type (capitula arrangement type).

Panicle was recovered as the ancestral state of *Artemisia* and for all the eight clades we identified (Fig. 3B; Supplementary data Fig. S13). It was the most common synflorescence type in *Artemisia* (85 % of the taxa sampled in the ingroup). Raceme was restricted to only a few lineages of Clade 1 (*A. norvegica*), Clade 6 [*A. rutifolia*, *A. furcata*, *A. capitata* (= *Sphaeromeria capitata*)] and [*A. macarthuri* (*= Sphaeromeria argentea*)] and nearly half of the species of Clade 7. Corymb was restricted to a single lineage in each of Clade 1 [*A. sibirica* (*=Filifolium sibiricum*) and *K. komarovii*] and Clade 6 (*A. macarthuri*). The shift from panicle to raceme or corymb occurred several times independently, mostly in the nodes near the tips (Fig. 3B; Supplementary data Fig. S13). The *Chrysanthemum*–*Ajania*–*Elachanthemum* clade shared the corymb synflorescence type. In the earliest-diverging group of *Artemisia*, *Ajania quercifolia* had a corymb synflorescence type and *Artemisiella stracheyi* a raceme.

#### Capitulum type (floret functional sex spatial arrangement in a capitulum).

Four types of capitula are reported in *Artemisia*, namely Type 1, heterogamous-disciform (capitula with outer florets female, central florets bisexual and fertile), receptacle glabrous; Type 2, heterogamous-disciform, receptacle pubescent; Type 3, heterogamous-disciform with central floret male, receptacle glabrous; and Type 4, homogamous-discoid (all florets bisexual and fertile), receptacle glabrous (Table 3; Fig. 3C). Type 1 was the ancestral and most common state (52 % of the taxa sampled in the ingroup) of *Artemisia*. The species of Clades 2, 3, 4 and 8 and the early-diverging lineages of Clade 1 all had Type 1 capitula. Type 2 (13 % of the taxa sampled in the ingroup) was restricted to Clades 5, 6 and 7 and dominated in Clade 7. Independent shifts from Type 2 to Type 1 occurred many times in Clade 7, such as in the lineages leading to *A. blepharolepis*, *A. shangnanensis* and *A. austriaca* (Fig. 3C; Supplementary data Fig. S14). Most species with Type 3 (16 % of the taxa sampled in the ingroup) occurred in Clade 1, except for two species (*A. porteri* and *A. filifolia*) with Type 3 in Clade 6. The shift from Type 1 to Type 4 occurred three times independently, in Clade 5, Clade 6 and in the lineage leading to *Artemisia juncea* in Clade 7. Interestingly, Clade 6 included all four capitulum types, implying that this lineage might successively have experienced a loss of receptacle hairs (Type 2 to Type 1), a loss of the female function in the bisexual florets (Type 1 to Type 3) and a loss of outer female florets (Type 3 to Type 4; Fig. 3C; Supplementary data Fig. S14).

#### Life form.

Perennial herb was the ancestral and most common state (64 % of the taxa sampled in the ingroup) of *Artemisia*, followed by shrubs or subshrubs (27 %) and annual or biennial herbs (9 %). The shrub life form originated independently at least ten times from the perennial herb life form. Apart from Clade 2 (all annual herbs) and Clade 8 (all perennial herbs), all clades included shrub species. Shrub life form predominated in Clade 4 and Clade 6 (Fig. 3D; Supplementary data Fig. S15). The annual life form had originated independently at least seven times from the perennial life form. Apart from Clades 6 and 8, all the other clades had annual or biennial herbs.

#### Basal leaf shape.

Pinnatisect leaves was the ancestral and most common state (79 % of the taxa sampled in the ingroup) of *Artemisia*. Entire or three-lobed leaves had originated at least six times. The species with entire or three-lobed leaves were clustered in Clades 1, 3, 6 and 8 (Fig. 3E; Supplementary data Fig. S16).

#### Basal leaf size.

Small leaves (basal leaf size <225 mm^2^) was the ancestral leaf size state of *Artemisia*. In this study, species of *Artemisia* with large, medium and small leaves accounted for 27, 42 and 31 % of the sampled taxa, respectively (Fig. 3F; Supplementary data Fig. S17). Large leaves were concentrated in Clades 2 and 8 and small leaves in Clades 5 and 6. The transition from small to medium leaves occurred three times (in Clades 1, 4 and 7). The shift from small to large leaves occurred four times, respectively, in one early-diverging lineage of Clade 1 (including *A. laciniata*), in the clade including *A. tournefortiana* in Clade 2, in the earliest-diverging lineage of Clade 5 (including *A. annua*) and in Clade 8 (Fig. 3F; Supplementary data Fig. S17). Secondary leaf size transition from large to medium occurred at least two times in Clades 5 and 8 (Fig. 3F).

## DISCUSSION

### Phylogeny of *Artemisia*

Nuclear genome SNPs were used for the first time to reconstruct the phylogeny of *Artemisia* (Fig. 2). It is well known that the nuclear genome SNP data obtained from genome skimming based on reference genomes has a high rate of missing data owing to low depth. Although the alleles in polyploids can be lost owing to unequal alignments between different alleles, the loss of alleles owing to low sequencing depth might be more frequent than the loss of loci owing to polyploidy (Olofsson *et al.*, 2019). Moreover, as another attempt to study the taxonomy and evolution of *Artemisia*, we obtained the transcriptome data of 100 species of *Artemisia* to test the robustness of the present phylogeny. The backbone of the topology obtained from the phylotranscriptomic analysis (unpublished data) was consistent with the present one based on nuclear genome SNPs. Therefore, we think that our current phylogeny based on SNPs is reliable.

**Fig. 4.**
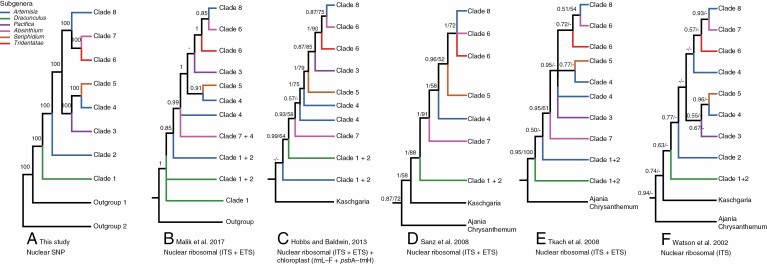
Comparison of *Artemisia* phylogenies. (A) simplified phylogenetic tree of our Fig. 2. Only eight clades and outgroups are shown. Numbers above branches indicate bootstrap percentages from the ML analysis. (B) Simplified phylogenetic tree of figure 1 from the paper by Malik *et al.* (2017). Numbers above branches indicate posterior probabilities from Bayesian analyses. (C) Simplified phylogenetic tree of figure 1 from the paper by Hobbs and Baldwin (2013). Numbers above branches indicate posterior probabilities from Bayesian analyses and bootstrap percentages from the ML analysis. (D) Simplified phylogenetic tree of figure 1 from the paper by Sanz *et al.* (2008). Numbers above branches indicate posterior probabilities from Bayesian analyses and parsimony bootstrap support. (E) Simplified phylogenetic tree of figure 1 from the paper by Tkach *et al.* (2008). Numbers above branches indicate posterior probabilities from Bayesian analyses and parsimony bootstrap support. (F) Simplified phylogenetic tree of figure 1 from the paper by Watson *et al.* (2002). Numbers above branches indicate posterior probabilities from Bayesian analyses and parsimony bootstrap support. The colours of branches indicate the traditional subgeneric taxonomy of *Artemisia*. Tip names of the simplified trees correspond to the names of the eight clades shown in Fig. 2.

We confirmed that the ‘*Dracunculus*’ and ‘*Seriphidium*’ lineages belonged to *Artemisia*. *Seriphidium* and *Tridentatae*, although both exhibiting a homogamous-discoid capitulum type, were shown to be two independent lineages, as suggested by Rydberg (1916) and McArthur *et al.* (1981). Their similarity in the capitulum type was the result of convergent evolution (McArthur *et al.*, 1981; McArthur and Sanderson, 1999; Shultz, 2009). Seven small or monotypic genera (*Crossostephium*, *Filifolium*, *Mausolea*, *Neopallasia*, *Picrothamnus*, *Turaniphytum* and *Sphaeromeria*) were shown to be nested in *Artemisia* and should be treated as members of *Artemisia*, as previously suggested (Watson *et al.*, 2002; Vallès *et al.*, 2003; Sanz *et al.*, 2008, 2011; Tkach *et al.*, 2008; Garcia *et al.*, 2011*a*, *b*; Pellicer *et al.*, 2011; Riggins and Seigler, 2012; Hobbs and Baldwin, 2013). We have summarized the major phylogenetic hypotheses focused on the whole genus *Artemisia* (Fig. 4B–E) and identified recurrent issues in previous phylogenies based on nuclear ribosomal DNA (nrDNA) or nrDNA and chloroplast DNA (cpDNA), such as the relationship between *Kaschgaria* and *Artemisia*, the relationships among the subgroups in *Artemisia*, and the mismatch between the molecular phylogenies published so far and the present infrageneric taxonomy system.

### Circumscription of *Artemisia*

*Kaschgaria* used to be considered as the sister group of the genus *Artemisia* (Fig. 4; Watson *et al.*, 2002; Sanz *et al.*, 2008; Hobbs and Baldwin, 2013). Our analyses showed that *K. komarovii* was nested in *Artemisia* and was sister to *A. salsoloides* (Fig. 2; Supplementary data Figs S1–S9); *K. komarovii* exhibited *Artemisia* pollen type and had the same hair type (stellate) on the corolla, life form (shrub) and leaf shape (entire or three-lobed) as its sister species *A. salsoloides* (Fig. 3; Pellicer *et al.*, 2011). Our results supported that *Kaschgaria* was not an independent genus. A similar topology but with low support was also obtained in two previous studies (Tkach *et al.*, 2008; Pellicer *et al.*, 2011). *Kaschgaria* was established by removing *Kaschgaria brachanthemoides* from *Artemisia* and *K. komarovii* from *Tanacetum* (Poljakov, 1957). Although only one species, *K. komarovii*, was sampled in our study, the monophyly of *Kaschgaria* was supported by previous phylogenies (Pellicer *et al.*, 2011). Based on our phylogenetic analyses, we propose to merge *Kaschgaria* with *Artemisia*, hence restoring the previously applied name, *Artemisia brachanthemoides* C. Winkl., and combining *K. komarovii* into *Artemisia* became necessary. Given that the specific epithet ‘*komarovii*’ was already used in *Artemisia*, here we propose a new name, ***Artemisia rubiginosa*** B.H. Jiao & T.G. Gao, **nom. nov.**; basionym *Tanacetum komarovii* Krasch. & N. Rubtz. (1946); synonym *Kaschgaria komarovii* (Krasch. & Rubtzov) Poljakov (1957). The specific epithet ‘*rubiginosa*’ refers to the reddish-brown colour of the margins of phyllaries.

Our results revealed, for the first time, the close relationship between *Ajania quercifolia* (*= Phaeostigma quercifolium*) and *Artemisiella stracheyi*. They formed a strongly supported clade (BS = 100 %; Fig. 2; Supplementary data Figs S1–S9), implying that the circumscription of the relatively recently established genus *Phaeostigma* (Muldashev, 1981; Huang *et al.*, 2017) needed to be revised. The clade consisting of the two species was sister to *Artemisia* (BS = 100 %; Fig. 2; Supplementary data Figs S1–S9). The result of our coalescence analysis, however, showed that *Artemisiella stracheyi* and *Ajania quercifolia* were nested in *Artemisia*, in independent subclades. Thus, their positions were unstable and not resolved (LPP = 0.64, 0.9; Supplementary data Fig. S10). Our results supported the close relationship of the *Ajania–Chrysanthemum* clade with *Artemisia* (Fig. 4; Watson *et al.*, 2002; Sanz *et al.*, 2008; Tkach *et al.*, 2008).

### Infrageneric taxonomy of *Artemisia*

The genus *Artemisia* consisted of eight highly supported clades (Figs 2, 4). Within *Artemisia*, Clade 1 (subgenus *Dracunculus* and some species of subgenus *Artemisia*) was the earliest-diverging lineage (Tkach *et al.*, 2008; Pellicer *et al.*, 2011; Malik *et al.*, 2017), followed by the newly discovered Clade 2. Clade 3 (subgenus *Pacifica*) was sister to Clade 4 + Clade 5, in contrast to the results presented in the latest study of the genus *Artemisia* (Malik *et al.*, 2017; Fig. 4B). Although the sister relationship between Clades 6 and 7 was strongly supported in our phylogeny (Fig. 2; BS = 100 %), it was not found in all our analyses. The ML trees based on lower missing data levels (Supplementary data Figs S2 and S3) and coalescence trees (Supplementary data Figs S10 and S11) showed that Clade 6 was sister to Clade 8, which was similar to the previous phylogenies (Fig. 4B–E; Watson *et al.*, 2002; Sanz *et al.*, 2008; Tkach *et al.*, 2008; Hobbs and Baldwin, 2013; Malik *et al.*, 2017). Therefore, although the respective monophyly of Clades 6, 7 and 8 was strongly supported, the relationships among them were not fully resolved, and further research is needed.

*Artemisia* subg. *Pacifica* (Clade 3) contains four species endemic to littoral habitats of Southeast Asia and littoral to subalpine habitats of the Hawaiian Islands (Hobbs and Baldwin, 2013). Although we sampled only one species, we are inclined to treat *A.* subg. *Pacifica* as a monophyletic group for the following three reasons. First, previous phylogenetic studies have showed that all the four species of *A.* subg. *Pacifica* formed a strongly supported monophyletic clade (Hobbs and Baldwin, 2013; Pellicer *et al.*, 2014). Second, all four species of this subgenus share many morphological characters, such as small shrubs, leaves clustered near tips, and achenes conspicuously five-ribbed and glandular. Third, the other three species not sampled are endemic to Hawaiian Islands, geographically far away from other species of *Artemisia*. Hobbs and Baldwin (2013) suggested that one long-distance dispersal event from Southeast Asia to the Hawaiian Islands could be responsible for the individualization of this subgenus. Thus, we think it is appropriate to treat this subgenus as monophyletic.

#### The first discovery of Clades 2 and 4.

All species in Clade 2, namely *A. hedinii*, *A. tournefortiana*, *A. biennis*, *A. baxoiensis* and *A. pectinata* (*= Neopallasia pectinata*), were also sampled in previous phylogenies (e.g. Malik *et al.*, 2017), but they usually had an isolated position in the tree or clustered with other species with low support (Fig. 4B, C, E, F; Jiao *et al.*, 2019). Our results showed, for the first time, the close relationship among these annual herbs. (Fig. 2). And the species belonging in Clade 4 formed a polyphyletic group with unresolved positions in previous studies (Tkach *et al.*, 2008; Malik *et al.*, 2017; Fig. 4B, E). Our phylogenomic results discovered a highly supported Clade 4, which was sister to Clade 5 (Fig. 2).

#### *The close relationship between the subgenus Tridentatae and some species of subgenus* Absinthium (A. lagocephala, A. rutifolia *and* A. younghusbandii*).*

Species of subgenus *Tridentatae* are endemic to the desert shrublands of western North America. Owing to insufficient sampling and markers without strong resolution power, the origin of the subgenus *Tridentatae* had remained mysterious for a long time. The subgenus *Tridentatae* used to be considered as closely related to subgenus *Seriphidium* because they both have homogamous capitula (Ling, 1991*a*), whereas other researchers suggested that they originated independently and that subgenus *Artemisia* is the ancestral stock for subgenus *Tridentatae* (McArthur *et al.*, 1981; Garcia *et al.*, 2011*b*). Our results showed that *A. lagocephala*, *A. rutifolia* and *A. younghusbandii* formed the earliest-diverging lineage of Clade 6, grouping with subgenus *Tridentatae*, in contrast to previous results. Malik *et al.* (2017), based on ITS + ETS, showed that the clade consisting of *A. lagocephala*, *A. rutifolia* and *A. younghusbandii* (PP = 0.94) was sister to some species here included in Clade 8 with medium support (PP = 85; Fig. 4 B). *Artemisia lagocephala*, *A. rutifolia* and *A. younghusbandii* were distributed mainly in the steppes or forest steppes of Northeastern Asia. The Beringian species *A. flava* and *A. furcata* formed an independent lineage sister to all the New World species in Clade 6 (Fig. 2). Our phylogeny suggested a possible scenario about the history of subgenus *Tridentatae*: the ancestors of subgenus *Tridentatae* dispersed from Eurasia to North America through Northeastern Asia (more specifically, through the Bering Land Bridge), then diversified in similar new habitats of western North America.

#### *Relationships among species and within each clade of* Artemisia.

In this study, the interspecific relationships in subgenera *Dracunculus* and *Seriphidium*, which probably underwent rapid radiation (Garcia *et al.*, 2011*b*; Pellicer *et al.*, 2011; Malik *et al.*, 2017), and in all the other clades were clearly resolved (Fig. 2). Some clades also showed distinct internal structures, and the positions of some taxonomically difficult taxa were resolved, e.g. the species of subgenus *Artemisia* in Clades 2 and 4; and the group of *A. lagocephala*, *A. rutifolia* and *A. younghusbandii*.

The core *Dracunculus* clade in Clade 1 was split into two lineages, one of them being the *A. dracunculus* lineage (= clade 2 in fig. 2 of the paper by Pellicer *et al.*, 2011). The phylogenetic placement of the two morphologically unique species, *Artemisia eriocarpa* (= *Mausolea eriocarpa*) and *Artemisia eranthema* (= *Turaniphytum eranthemum*), was fully resolved, for the first time. They were sister to each other, and this two-species lineage diverged after the *A. dracunculus* complex lineage (Fig. 2; Clade 1, BS = 100 %).

Clade 5 was composed of three highly supported lineages, corresponding to the three subgenera *Seriphidium*, *Absinthium* and *Artemisia*, in coherence with previous results (Pellicer *et al.*, 2014; Malik *et al.*, 2017). Our results suggested that subgenus *Artemisia* (including *A. annua* and *A. carvifolia*) was the earliest-diverging lineage, and that subgenus *Absinthium* (including *A. anethifolia*, *A. anethoides*, *A. zhaodongensis* and *A. nakaii*) was sister to subgenus *Seriphidium* (Fig. 2; Supplementary data Fig. S10).

The monophyly of Clade 8 was proposed in the previous phylogenies (Hobbs and Baldwin, 2013; Malik *et al.*, 2017; Fig. 4). Our new phylogeny strongly supported Clade 8 as monophyletic (BS = 84 %; LPP = 1). Furthermore, we newly identified three main lineages within the latter clade (Fig. 2, Clade 8a–c, BS = 100 %).

### Conflicts between phylogenies and the traditional taxonomy of *Artemisia*

Previous studies revealed conflicts between phylogenies and the traditional taxonomy of *Artemisia* to some extent (Watson *et al.*, 2002; Hobbs and Baldwin, 2013; Malik *et al.*, 2017). Our new analyses revealed that five of the six subgenera of *Artemisia* previously recognized were not supported as monophyletic. The only exception is the subgenus *Pacifica* (Fig. 2). We here compared our eight-clade phylogenetic framework (Fig. 2) with the infrageneric taxonomy including six subgenera (Fig. 4; Table 2). The subgenus *Artemisia* was shown to be polyphyletic (Fig. 2). With the exception of Clade 3 (including only subgenus *Pacifica*), all the other seven clades included some species of subgenus *Artemisia* (Fig. 2). Among them, Clade 8 and two newly discovered clades, Clades 2 and 4, were all composed of the species of subgenus *Artemisia*. Clade 1 was dominated by the species of subgenus *Dracunculus*, also including some species from subgenus *Artemisia*. Clade 5 was dominated by the species of subgenus *Seriphidium*, also including species from subgenera *Absinthium* and *Artemisia*. Clade 6 was dominated by species of subgenus *Tridentatae*, also including species from subgenera *Absinthium* and *Artemisia*. Clade 7 was dominated by the species of subgenus *Absinthium*, including species from subgenera *Seriphidium* and *Artemisia*. The phylogenetic framework and the evolutionary patterns of morphological characters can be used as an important reference for updating the subgeneric taxonomy of *Artemisia*.

### Morphological evolution within *Artemisia*

#### Pollen type.

The *Artemisia* pollen type, characterized by weakly ornamented pollen grains, was the ancestral state of all the sampled species of *Artemisia* and of *Ajania quercifolia* (Fig. 3; Supplementary data Fig. S12). All the sampled *Artemisia* species and all segregated genera nested in *Artemisia* shared this feature. This state was derived from the state ‘*Anthemis* pollen type’, corresponding to more ornamented, echinate pollen grains, which was displayed by most members of the tribe Anthemideae. Besides, *Elachanthemum* also experienced a transition from the *Anthemis* pollen type to the *Artemisia* pollen type (Fig. 3A; Supplementary data Fig. S12). *Artemisia* pollen type was highly consistent among *Artemisia* species and could be used to circumscribe the genus *Artemisia* (Fig. 3A; Sanz *et al.*, 2008), indicating that natural selection might have had a strong force on this character, which is associated with the anemophilous pollination mode.

#### Synflorescence type.

Synflorescence type was diagnostic for *Artemisia*, although the consistency of this character was slightly lower than for the pollen type. Panicle was the most common synflorescence type in *Artemisia* and was recovered as the ancestral state of the genus. The panicle state originated from an ancestor with corymbose synflorescence. Synflorescence type had been used for taxonomic purposes, particularly to define generic limits. Many genera were described based mainly on synflorescence type (e.g. *Crossostephium*, *Filifolium* and *Picrothamnus*). According to recent molecular phylogenetic studies, these genera were reduced into *Artemisia* (Watson *et al.*, 2002; Sanz *et al.*, 2008; Hobbs and Baldwin, 2013). In our phylogeny, species with a corymbose synflorescence type were all nested in Clade 1, whereas species with a racemose synflorescence type were all nested in Clades 6 and 7 (Fig. 3B; Supplementary data Fig. S13). Racemes, like compressed corymbs, represent the reduction trend in the evolution of *Artemisia* synflorescences and occurred mostly in the shallow nodes of the *Artemisia* phylogenetic tree, which could be regarded as local adaptations. We also noticed that the species in question were mostly distributed in high-elevation regions (e.g. *A. umbelliformis* and *A. pedemontana*) or extremely arid regions [*A. macarthuri* (*= Sphaeromeria argentea*) and *A. spinescens* (*= Picrothamnus desertorum*)]. Species displaying the panicle synflorescence type, contrary to the species with racemose synflorescences, seemingly were not able to adapt to habitats with low temperatures or lack of water. Therefore, reduction of branching in synflorescences seemed to have been an adaptation of *Artemisia* species to extreme habitats.

#### Capitulum type.

Many *Artemisia* species and many allies of *Artemisia* displayed the same capitulum type (Type 1; Table 3; Fig. 3C), hence this character could not be used to define the genus *Artemisia*. However, capitulum type was the most important character for its infrageneric taxonomy (Fig. 3C; Poljakov, 1961*a*; Tutin *et al.*, 1976; Shultz, 2006; Ling *et al.*, 2011). The four capitulum types used in this study corresponded well to Besser’s taxonomy into four sections (Besser, 1829, 1832, 1834, 1835; Table 1). The current taxonomy of *Artemisia* including six subgenera was based exclusively on two traits, capitulum type and distribution (Table 2). This taxonomy, however, did not correspond well to the clades recovered in our phylogeny, in which five of the six subgenera were shown to be para- or polyphyletic (Fig. 2). Our analysis (Fig. 3C; Supplementary data Fig. S14) highlighted the high plasticity of capitulum type in *Artemisia*. For instance, Clade 6 included all four types of capitula (Fig. 3C; Supplementary data Fig. S14). The evolutionary history of capitulum type is much more complicated than previously thought (e.g. Ling, 1982), and this trait alone could not be used to classify taxa within *Artemisia*.

#### Life form.

Perennial herb was reconstructed as the ancestral life form state of *Artemisia* and also the ancestral state of its allies (Fig. 3D). This state had shifted independently to annual herb and shrub seven and ten times, respectively, indicating that the life form of *Artemisia* has been highly labile (Fig. 3D). A similar trend was observed in other taxa of Asteraceae (Beaulieu *et al.*, 2013; Jara-Arancio *et al.*, 2018; Andrés-Sánchez *et al.*, 2019). Some studies indicated that there might be only a few genes involved in controlling the transition from herbaceous to woody in Asteraceae (Groover, 2005; Lens *et al.*, 2012). Therefore, such frequent habit changes in *Artemisia* could be understood as adaptations to special habitats. In general, among the eight clades, five were dominated by a single type of life form. For example, Clade 2 consisted of annual herbs, whereas Clades 3, 4 and 6 included mostly shrubs, and Clade 1 consisted mostly of perennial herbs. Therefore, the life form could still be considered a useful character in the infrageneric taxonomy of *Artemisia*.

#### Leaf shape.

The ancestral leaf state of both *Artemisia* and the other Anthemideae genera was reconstructed as pinnatisect leaves (Fig. 3E; Supplementary data Fig. S16). Some species with entire or three-lobed leaves were reported in *Artemisia*, e.g. *A. dracunculus* and *A. ludoviciana*. This leaf shape state evolved independently at least six times. Despite multiple origins, the distribution of this character state was not random and was concentrated in some lineages, such as the *A. dracunculus* complex clade in Clade 1, most species in Clade 6 and the whole of Clade 8a (Fig. 3E). Therefore, leaf shape could be used to assist in the infrageneric taxonomy of *Artemisia*. Increasing the number of leaf lobes could help plants dissipate heat in hot environments (Vogel, 1968). Many *Artemisia* species were widely distributed in dry areas with a hot growing season. We speculated that species with pinnatisect leaves might have higher fitness in these dry and hot areas. In contrast, most *Artemisia* species with a low number of leaf lobes grew in relatively closed and humid environments, such as forest margins and riverbanks (e.g. *A. viridissima*), where heat dissipation was no longer a strong selection factor.

#### Leaf size.

The ancestral leaf size of *Artemisia* was reconstructed as small. The transition from small to medium leaves occurred three times, and four transitions to large leaves were inferred (Fig. 3F). Leaf size also reflected the adaptation of the plant to the environment. Smaller leaves were beneficial to reduce leaf temperature instantly and avoid heat damage (Vogel, 1970), thus *Artemisia* species with smaller leaves could be better adapted to dry and hot environments. Most species with large leaves grew in relatively humid environments, such as forest margins and riverbanks, such as most species in Clade 8. Although leaf size had a relatively high environmental plasticity, it still had diagnostic value for the infrageneric taxonomy of *Artemisia*. For example, most species in Clade 6 possessed small leaves, and most species in Clade 8 displayed large leaves.

Although the evolutionary history of capitulum types was more complex than previously thought (e.g. Ling, 1982), and despite the fact that the subgeneric taxonomy based on this trait did not agree well with our molecular phylogeny (Fig. 2) and the other molecular phylogenies (Watson *et al.*, 2002; Hobbs and Baldwin, 2013; Malik *et al.*, 2017), it was still the most important character in the infrageneric taxonomy of *Artemisia*. Other characters, such as life form, leaf shape and leaf size, were consistent in large or small lineages within *Artemisia*. For instance, all the species in Clade 2 were annual herbs, all the species in Clade 6 were shrubs, and most species in Clade 8 had large leaves (Fig. 3). Therefore, given that the monophyletic subgenera could not be established based only on capitulum type and geographical distribution, the addition of more characters, such as life type and leaf shape, could be beneficial for updating the subgeneric taxonomy of *Artemisia*.

### Revised infrageneric taxonomy of *Artemisia*

The present phylogenetic framework and morphological evolution patterns revealed in this study showed that the existing infrageneric taxonomy of *Artemisia* consisting of six subgenera did not reflect the phylogenetic relationships well. The eight-clade phylogenetic framework could be used as the basis for updating the infrageneric taxonomy of *Artemisia*. Here, we propose a new framework for the infrageneric taxonomy of *Artemisia*, with eight recognized subgenera to accommodate the new results. Considering that each morphological character individually is not enough to circumscribe the eight subgenera, we have provided a table to compare the taxonomic relevance of the different morphological character combinations (Table 4). Given that the focus of the present research is the framework of the infrageneric taxonomy of *Artemisia*, not the taxonomic details of each of its eight subgenera, we will discuss these in other papers and books.

**Table 4.** Morphological comparison of the eight subgenera of *Artemisia*

Subgenus	Synflorescence type	Receptacle hair	Outer female florets	Central florets	Life form	Basal leaf morphology	Basal leaf size
*Dracunculus*	Panicle, rarely raceme or corymb	Absent	Present	Hermaphrodite, but female sterile	Perennial herb or shrub, rarely annual herb	Pinnatisect, rarely entire or three-lobed	Medium, rarely big or small
*Pectinatae*	Panicle	Absent	Present	Hermaphrodite	Annual herb	Pinnatisect	Big or small
*Pacifica*	Panicle	Absent	Present	Hermaphrodite	Shrub	Pinnatisect	Small
*Ponticae*	Panicle	Absent	Present	Hermaphrodite	Perennial herb or shrub	Pinnatisect	Medium, rarely big or small
*Seriphidium*	Panicle	Present	Absent	Hermaphrodite	Perennial herb, rarely annual herb or shrub	Pinnatisect	Small, rarely medium
*Tridentatae*	Panicle, rarely raceme	Present	Absent	Hermaphrodite	Shrub, rarely perennial herb	three-lobed, rarely pinnatisect	Small, rarely medium
*Absinthium*	Panicle, rarely raceme	Present	Present	Hermaphrodite	Perennial herb, rarely annual herb or shrub	Pinnatisect	Small or medium, rarely big
*Artemisia*	Panicle	Absent	Present	Hermaphrodite	Perennial herb	Pinnatisect, rarely entire or three-lobed	Big or medium

#### *Artemisia* L. subgenus *Dracunculus* (Besser) Rydb.

Perennial herbs or shrubs, occasionally annual herbs; leaves of various shapes and sizes; synflorescence panicle, rarely raceme or corymb; capitula heterogamous-disciform with central florets male (Type 3) or heterogamous-disciform (Type 1).

It corresponds to Clade 1 revealed in this study (Fig. 2). It is used to accommodate the expanded subgenus *Dracunculus* (Besser) Rydb., including the former subgenus *Dracunculus* and some herbaceous species of the former subgenus *Artemisia*.

#### *Artemisia* L. subgenus *Pectinata* B.H. Jiao & T.G. Gao subg.nov.

Annuals or biennials; leaves pinnatisect, large or small; synflorescence panicle; capitula heterogamous-disciform (Type 1).

It corresponds to Clade 2 revealed in this study (Fig. 2). We propose to treat this clade as a new subgenus, ***Artemisia*** subgenus ***Pectinatae*** B.H. Jiao & T.G. Gao **subg. nov.** TYPE: *Artemisia pectinata* Pall.

#### *Artemisia* L. subgenus *Pacifica* C.R. Hobbs & B.G. Baldwin

Shrubs; leaves pinnatisect, large; synflorescence panicle; capitula heterogamous-disciform (Type 1).

It corresponds to Clade 3 revealed in this study (Fig. 2).

#### *Artemisia* L. subgenus *Ponticae* B.H. Jiao & T.G. Gao subg. nov.

Subshrubs or shrubs; leaves pinnatisect, large; synflorescence panicle; capitula heterogamous-disciform (Type 1).

It corresponds to Clade 4 revealed in this study (Fig. 2).

We propose to treat this clade as a new subgenus, ***Artemisia*** subgenus ***Ponticae*** B.H. Jiao & T.G. Gao **subg. nov.** TYPE: *Artemisia pontica* L.

#### *Artemisia* L. subgenus *Seriphidium* Besser ex Less.

Annuals, biennials, perennials or subshrubs; leaves pinnatisect, leaf size varies from small to large; synflorescence panicle; capitula heterogamous-disciform (Type 1), heterogamous-disciform, receptacle pubescent (Type 2) or homogamous-discoid (Type 4).

It corresponds to Clade 5 revealed in this study (Fig. 2). It is used to accommodate the expanded subgenus *Seriphidium* Besser ex Less., including the former subgenus *Seriphidium* (except *A. juncea* group) and annual herbaceous species of the former subgenus *Artemisia* (*A. annua* and *A. carvifolia*) and subgenus *Absinthium* (*A. anethifolia*, *A. anethoides* and *A. nakaii*).

#### *Artemisia* L. subgenus *Tridentatae* (Rydb.) McArthur

Shrubs or subshrubs; leaves entire or three-lobed, rarely pinnatisect, leaves small; synflorescence panicle, raceme or corymb; capitula heterogamous-disciform (Type 1), heterogamous-disciform, receptacle pubescent (Type 2), heterogamous-disciform with central florets male (Type 3) or homogamous-discoid (Type 4).

It corresponds to Clade 6 revealed in this study (Fig. 2). It is used to accommodate the expanded subgenus *Tridentatae* (Rydb.) McArthur, including all species of the former subgenus *Tridentatae*, the Northeastern Asian species of subgenus *Absinthium* (*A. lagocephala*, *A. rutifolia* and *A. younghusbandii*) and some species of subgenus *Artemisia* from the Beringian and the New World.

#### *Artemisia* L. subgenus *Absinthium* (Mill.) Less.

Annuals, biennials, perennials or subshrubs; leaves pinnatisect, medium; synflorescence panicle or raceme; capitula heterogamous-disciform, receptacle pubescent (Type 2) or heterogamous-disciform (Type 1).

It corresponds to Clade 7 revealed in this study (Fig. 2). It is used to accommodate the redefined *Artemisia* subgenus *Absinthium* (Mill.) Less., including the *A. juncea* group and *A. blepharolepis*, excluding *A. anethifolia*, *A. anethoides*, *A. nakaii*, *A. lagocephala*, *A. rutifolia* and *A. younghusbandii*.

#### *Artemisia* L. subgenus *Artemisia*

Perennial herbs; leaves pinnatisect, occasionally entire or three-lobed, large, occasionally medium; synflorescence panicle; capitula heterogamous-disciform (Type 1).

It corresponds to Clade 8 revealed in this study (Fig. 2). It is used to accommodate the redefined *Artemisia* subgenus *Artemisia*, excluding the species now belonging to subgenera *Dracunculus*, *Pectinatae*, *Ponticae*, *Seriphidium*, *Tridentatae* and *Absinthium*.

The broad sampling and the strong phylogenetic resolution obtained in our study warrant that this new subgeneric taxonomy is robust and should last over time. However, it is important to be aware that the addition of further species that could be analysed within the same phylogenetic framework (e.g. using the same nuclear SNPs data set) would allow the systematic value of these morphological characters to be confirmed and/or refined.

## Conclusions

Overall, we revealed eight highly supported clades in *Artemisia* and suggested that the genus *Kaschgaria* should be merged into *Artemisia*. The morphological characters traditionally used for the infrageneric taxonomy of *Artemisia* do not match the new phylogenetic tree. They originated independently more than once and could not be used alone to define the eight clades revealed in the new phylogeny. We proposed a revised framework for the subgeneric taxonomy of *Artemisia* to accommodate the new results. These results laid a foundation for further systematic and evolutionary studies on *Artemisia* and extensive utilization of its rich biodiversity resources.

## Supplementary Material

mcad051_suppl_Supplementary_Figures

mcad051_suppl_Supplementary_Table_S1

mcad051_suppl_Supplementary_Table_S2

## SUPPLEMENTARY DATA

Supplementary data are available online at https://academic.oup.com/aob and consist of the following. Table S1: taxa included in this study. Table S2: the number of single nucleotide polymorphisms obtained from different missing levels. Figure S1: maximum likelihood phylogenetic tree obtained from the alignment of nuclear single nucleotide polymorphisms allowing for >50 % missing data per marker obtained by mapping low-depth whole-genome sequencing data reads to the *Artemisia annua* simplified coding sequences. Figure S2: maximum likelihood phylogenetic tree obtained from the alignment of nuclear single nucleotide polymorphisms allowing for >60 % missing data per marker obtained by mapping low-depth whole-genome sequencing data reads to the *Artemisia annua* simplified coding sequences. Figure S3: maximum likelihood phylogenetic tree obtained from the alignment of nuclear single nucleotide polymorphisms allowing for >70 % missing data per marker obtained by mapping low-depth whole-genome sequencing data reads to the *Artemisia annua* simplified coding sequences. Figure S4: maximum likelihood phylogenetic tree obtained from the alignment of nuclear single nucleotide polymorphisms allowing for >90 % missing data per marker obtained by mapping low-depth whole-genome sequencing data reads to the *Artemisia annua* simplified coding sequences. Figure S5: maximum likelihood phylogenetic tree obtained from the alignment of nuclear single nucleotide polymorphisms allowing for >50 % missing data per marker obtained by mapping low-depth whole-genome sequencing data reads to the *Chrysanthemum seticuspe* simplified coding sequences. Figure S6: maximum likelihood phylogenetic tree obtained from the alignment of nuclear single nucleotide polymorphisms allowing for >60 % missing data per marker obtained by mapping low-depth whole-genome sequencing data reads to the *Chrysanthemum seticuspe* simplified coding sequences. Figure S7: maximum likelihood phylogenetic tree obtained from the alignment of nuclear single nucleotide polymorphisms allowing for >70 % missing data per marker obtained by mapping low-depth whole-genome sequencing data reads to the *Chrysanthemum seticuspe* simplified coding sequences. Figure S8: maximum likelihood phylogenetic tree obtained from the alignment of nuclear single nucleotide polymorphisms allowing for >80 % missing data per marker obtained by mapping low-depth whole-genome sequencing data reads to the *Chrysanthemum seticuspe* simplified coding sequences. Figure S9: maximum likelihood phylogenetic tree obtained from the alignment of nuclear single nucleotide polymorphisms allowing for >90 % missing data per marker obtained by mapping low-depth whole-genome sequencing data reads to the *Chrysanthemum seticuspe* simplified coding sequences. Figure S10: multigene coalescent species tree estimated from 540 nuclear gene trees based on single nuclear polymorphism alignments obtained with *Artemisia annua* simplified coding sequences as reference using ASTRAL v.5.6.3. Figure S11: multigene coalescent species tree estimated from 540 nuclear gene trees based on single nuclear polymorphism alignments obtained with *Chrysanthemum seticuspe* simplified coding sequences as reference using ASTRAL v.5.6.3. Figure S12: evolution of pollen type in *Artemisia* and allies, showing the ancestral character traits implemented in RASP v.3.2 using the maximal likelihood method based on the new inferred tree in Fig. 2. Figure S13: evolution of synflorescence type in *Artemisia* and allies, showing the ancestral character traits implemented in RASP v.3.2 using the maximal likelihood method based on the new inferred tree in Fig. 2. Figure S14: evolution of capitulum type in *Artemisia* and allies, showing the ancestral character traits implemented in RASP v.3.2 using the maximal likelihood method based on the new inferred tree in Fig. 2. Figure S15: evolution of life form in *Artemisia* and allies, showing the ancestral character traits implemented in RASP v.3.2 using the maximal likelihood method based on the new inferred tree in Fig. 2. Figure S16: evolution of basal leaf form in *Artemisia* and allies, showing the ancestral character traits implemented in RASP v.3.2 using the maximal likelihood method based on the new inferred tree in Fig. 2. Figure S17: evolution of basal leaf size in *Artemisia* and allies, showing the ancestral character traits implemented in RASP v.3.2 using the maximal likelihood method based on the new inferred tree in Fig. 2.
