# Supplementary material for: Phylogenomics and morphological evolution of the mega-diverse genus Artemisia (Asteraceae: Anthemideae): implications for its circumscription and infrageneric taxonomy
Source: Ann Bot. 2023 Mar 28;131(5):867–83. doi: 10.1093/aob/mcad051 (PMC10184459; doi:10.1093/aob/mcad051)
Supplement: mcad051_suppl_Supplementary_Figures [file mcad051_suppl_supplementary_figures.pdf]

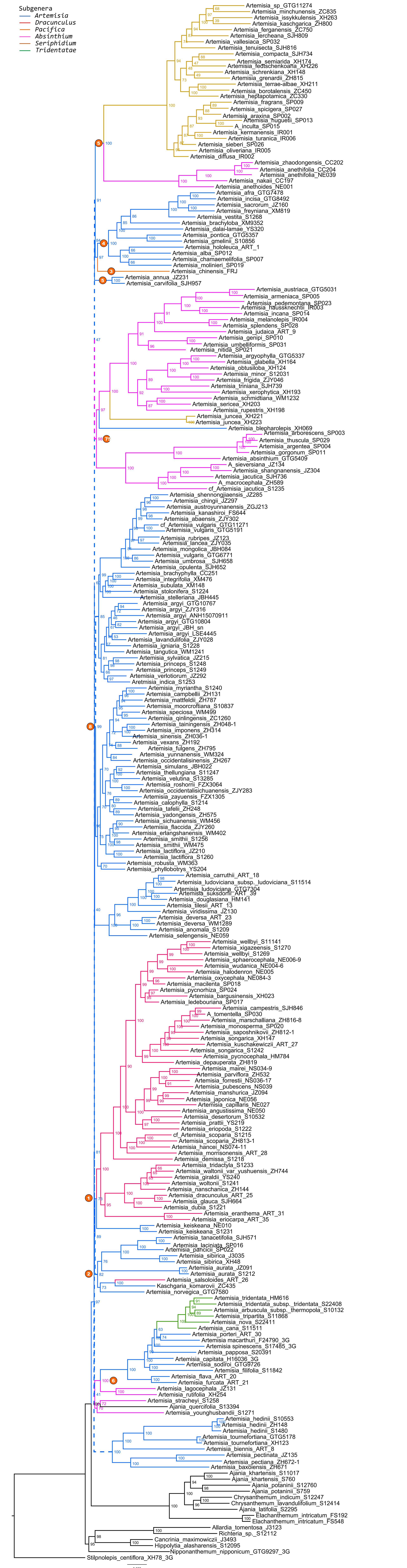

Fig. S1 Maximum likelihood phylogenetic tree obtained from the alignment of nuclear single nucleotide polymorphisms (SNPs) allowing for more than 50% missing data per marker obtained by mapping low depth whole genome sequencing data reads to the *Artemisia annua* simplified coding sequences (CDS). Nodes support was evaluated with 100 bootstrap replicates, bootstrap values (BS) are indicated along branches. The colors of branches indicate the traditional subgeneric classification of *Artemisia*. Dashed line denotes differences to the topology obtained with SNP alignments allowing for more than 80% missing data at each marker after mapping to *Artemisia annua* genome (Figure 1).

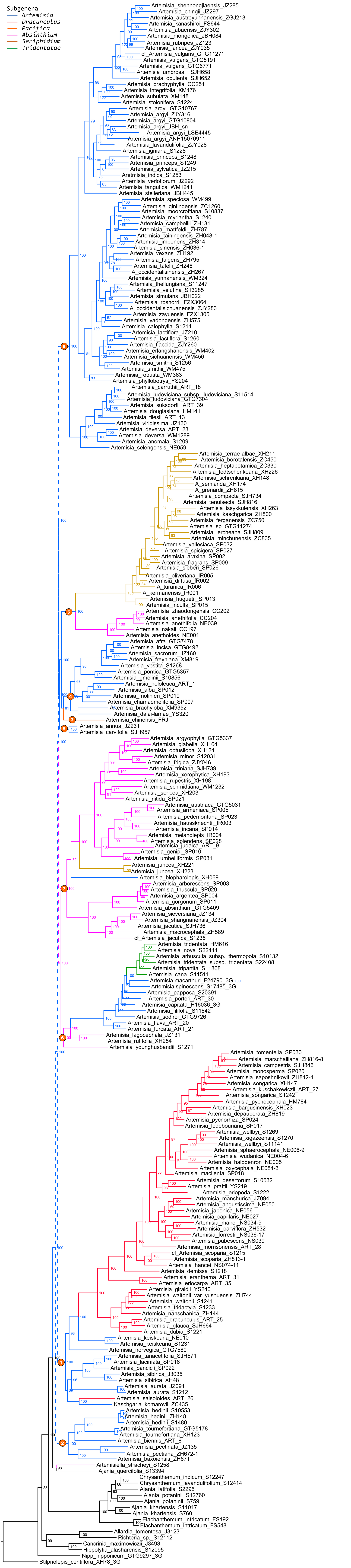

Fig. S2 Maximum likelihood phylogenetic tree obtained from the alignment of nuclear single nucleotide polymorphisms (SNPs) allowing for more than 60% missing data per marker obtained by mapping low depth whole genome sequencing data reads to the *Artemisia annua* simplified coding sequences (CDS). Nodes support was evaluated with 100 bootstrap replicates, bootstrap values (BS) are indicated along branches. The colors of branches indicate the traditional subgeneric classification of *Artemisia*. Dashed line denotes differences to the topology obtained with SNP alignments allowing for more than 80% missing data at each marker after mapping to *Artemisia annua* genome (Figure 1).

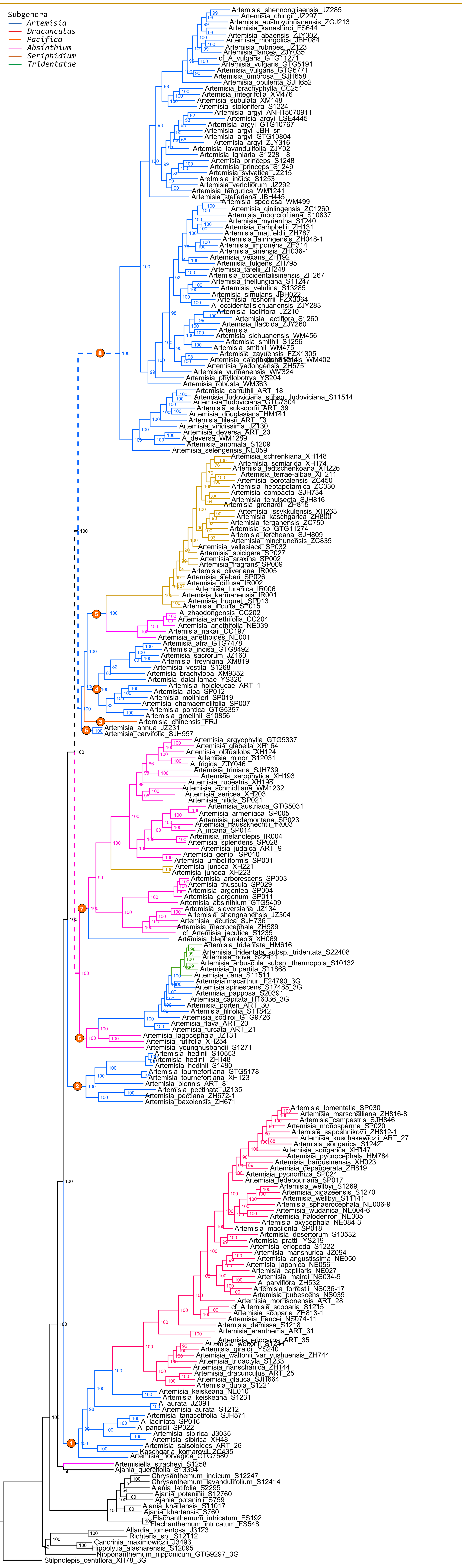

Fig. S3 Maximum likelihood phylogenetic tree obtained from the alignment of nuclear single nucleotide polymorphisms (SNPs) allowing for more than 70% missing data per marker obtained by mapping low depth whole genome sequencing data reads to the *Artemisia annua* simplified coding sequences (CDS). Nodes support was evaluated with 100 bootstrap replicates, bootstrap values (BS) are indicated along branches. The colors of branches indicate the traditional subgeneric classification of *Artemisia*. Dashed line denotes differences to the topology obtained with SNP alignments allowing for more than 80% missing data at each marker after mapping to *Artemisia annua* genome (Figure 1).

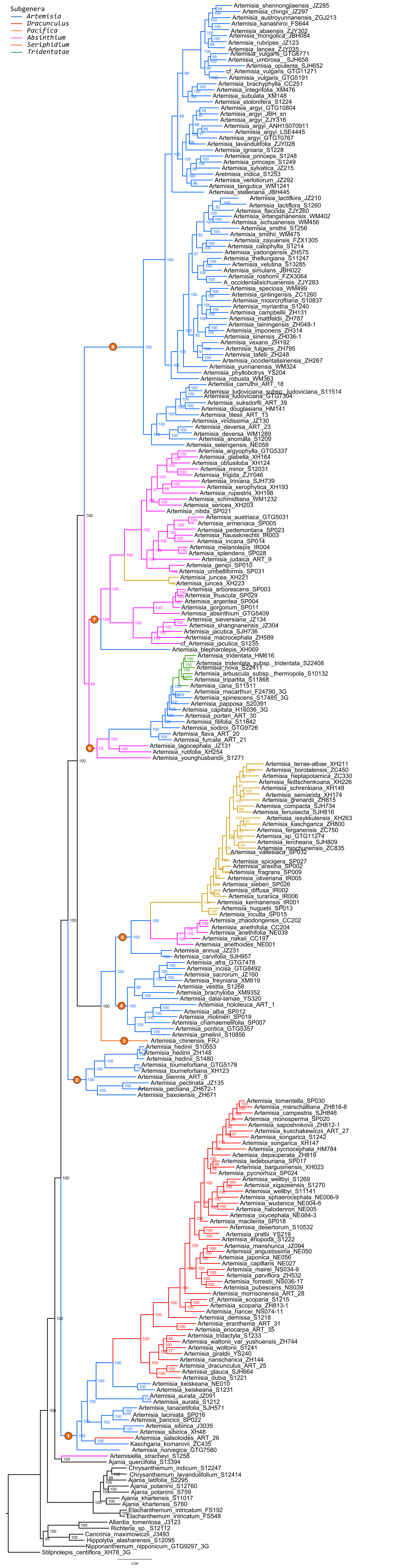

Fig. S4 Maximum likelihood phylogenetic tree obtained from the alignment of nuclear single nucleotide polymorphisms (SNPs) allowing for more than 90% missing data per marker obtained by mapping low depth whole genome sequencing data reads to the *Artemisia annua* simplified coding sequences (CDS). Nodes support was evaluated with 100 bootstrap replicates, bootstrap values (BS) are indicated along branches. The colors of branches indicate the traditional subgeneric classification of *Artemisia*. Dashed line denotes differences to the topology obtained with SNP alignments allowing for more than 80% missing data at each marker after mapping to *Artemisia annua* genome (Figure 1).

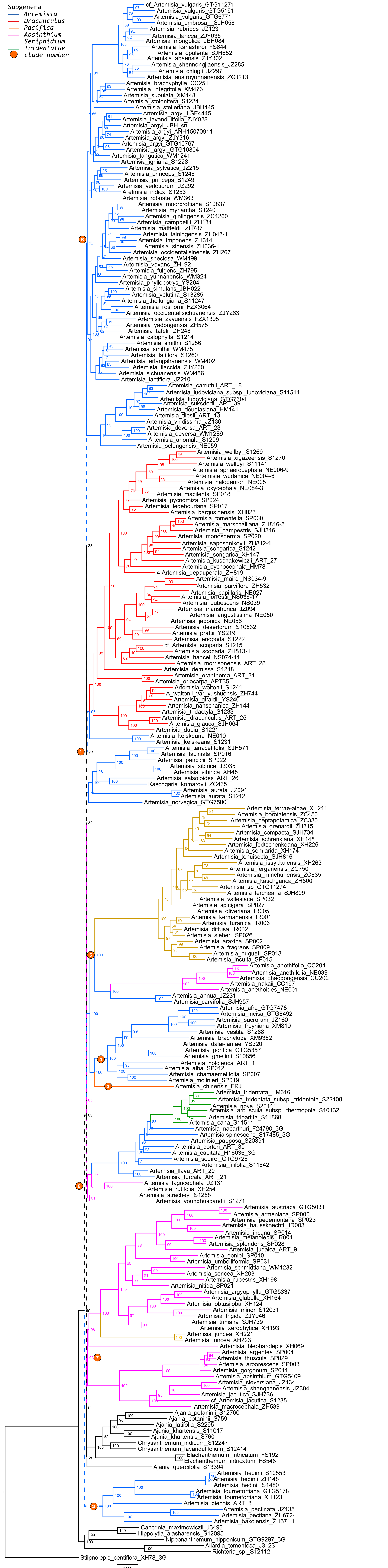

Fig. S5 Maximum likelihood phylogenetic tree obtained from the alignment of nuclear single nucleotide polymorphisms (SNPs) allowing for more than 50% missing data per marker obtained by mapping low depth whole genome sequencing data reads to the *Chrysanthemum seticuspe* simplified coding sequences (CDS). Nodes support was evaluated with 100 bootstrap replicates, bootstrap values (BS) are indicated along branches. The colors of branches indicate the traditional subgeneric classification of *Artemisia*. Dashed line denotes differences to the topology obtained with SNP alignments allowing for more than 80% missing data at each marker after mapping to *Artemisia annua* genome (Figure 1).

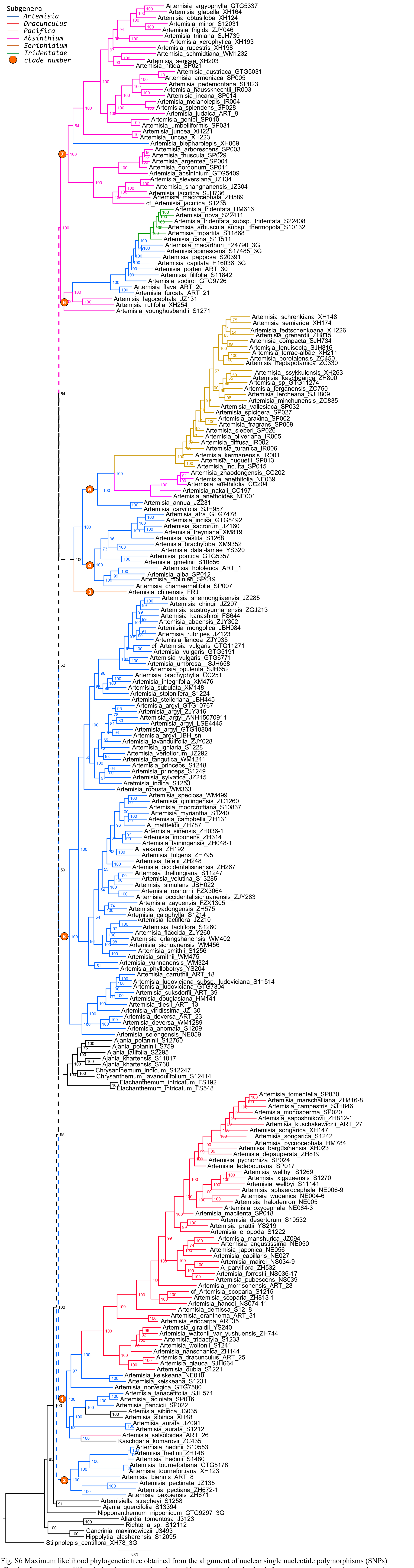

Fig. S6 Maximum likelihood phylogenetic tree obtained from the alignment of nuclear single nucleotide polymorphisms (SNPs) allowing for more than 60% missing data per marker obtained by mapping low depth whole genome sequencing data reads to the *Chrysanthemum seticuspe* simplified coding sequences (CDS). Nodes support was evaluated with 100 bootstrap replicates, bootstrap values (BS) are indicated along branches. The colors of branches indicate the traditional subgeneric classification of *Artemisia*. Dashed line denotes differences to the topology obtained with SNP alignments allowing for more than 80% missing data at each marker after mapping to *Artemisia annua* genome (Figure 1).

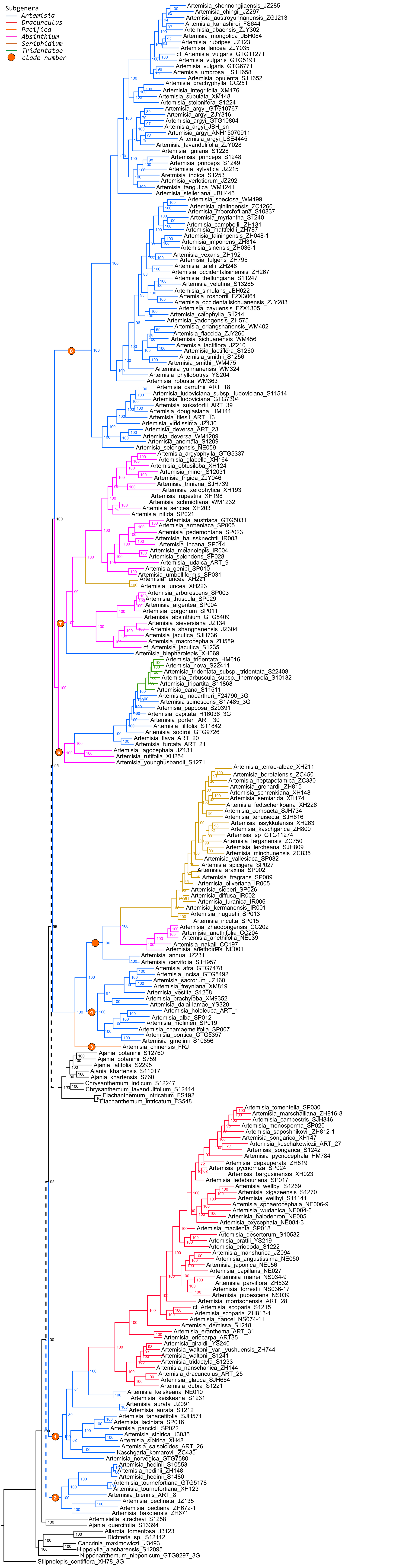

Fig. S7 Maximum likelihood phylogenetic tree obtained from the alignment of nuclear single nucleotide polymorphisms (SNPs) allowing for more than 70% missing data per marker obtained by mapping low depth whole genome sequencing data reads to the *Chrysanthemum seticuspe* simplified coding sequences (CDS). Nodes support was evaluated with 100 bootstrap replicates, bootstrap values (BS) are indicated along branches. The colors of branches indicate the traditional subgeneric classification of *Artemisia*. Dashed line denotes differences to the topology obtained with SNP alignments allowing for more than 80% missing data at each marker after mapping to *Artemisia annua* genome (Figure 1).

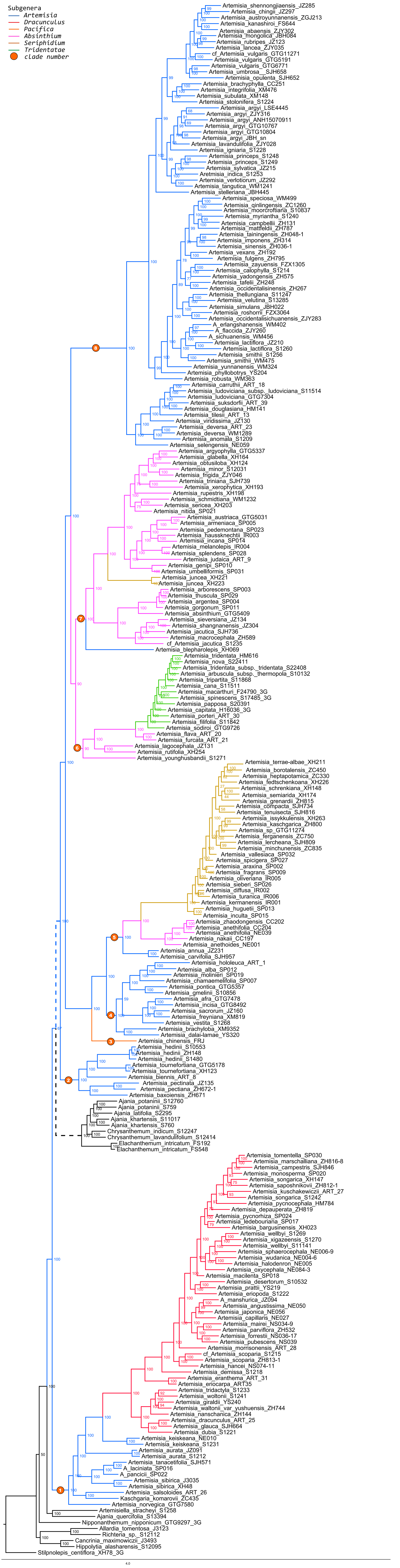

Fig. S8 Maximum likelihood phylogenetic tree obtained from the alignment of nuclear single nucleotide polymorphisms (SNPs) allowing for more than 80% missing data per marker obtained by mapping low depth whole genome sequencing data reads to the *Chrysanthemum seticuspe* simplified coding sequences (CDS). Nodes support was evaluated with 100 bootstrap replicates, bootstrap values (BS) are indicated along branches. The colors of branches indicate the traditional subgeneric classification of *Artemisia*. Dashed line denotes differences to the topology obtained with SNP alignments allowing for more than 80% missing data at each marker after mapping to *Artemisia annua* genome (Figure 1).

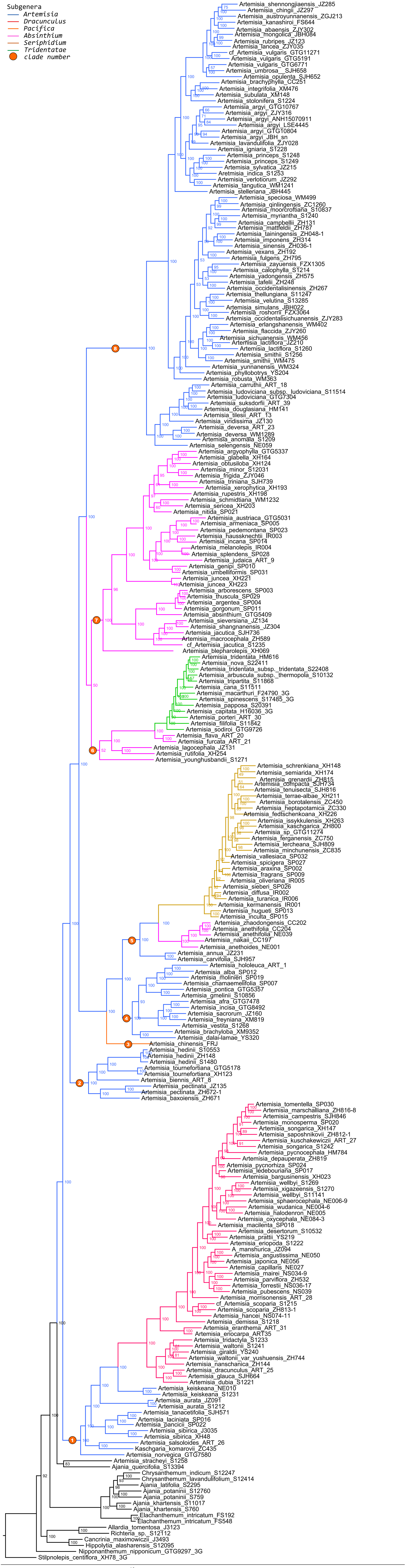

Fig. S9 Maximum likelihood phylogenetic tree obtained from the alignment of nuclear single nucleotide polymorphisms (SNPs) allowing for more than 90% missing data per marker obtained by mapping low depth whole genome sequencing data reads to the *Chrysanthemum seticuspe* simplified coding sequences (CDS). Nodes support was evaluated with 100 bootstrap replicates, bootstrap values (BS) are indicated along branches. The colors of branches indicate the traditional subgeneric classification of *Artemisia*. Dashed line denotes differences to the topology obtained with SNP alignments allowing for more than 80% missing data at each marker after mapping to *Artemisia annua* genome (Figure 1).

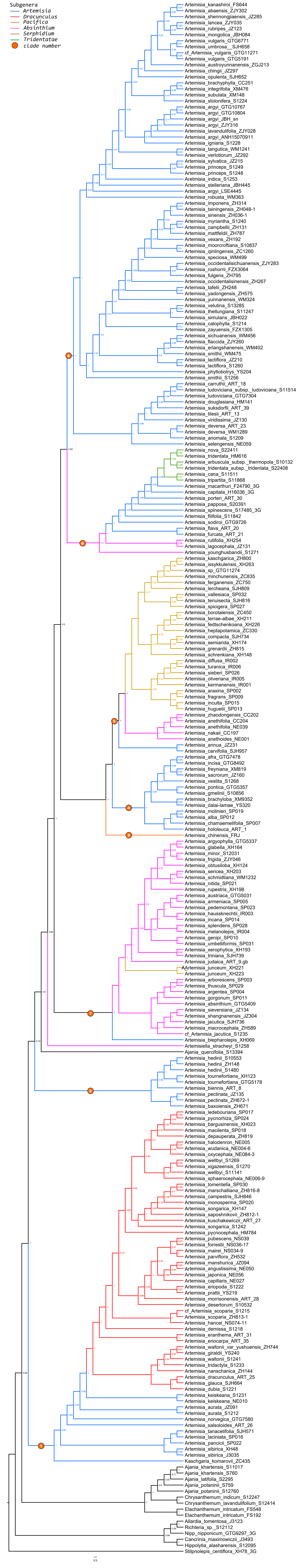

Fig. S10 Multigene coalescent species tree estimated from 540 nuclear gene trees based on single nuclear polymorphism (SNP) alignments (> 150 bp and containing ≥50% of the total number of samples) obtained with *Artemisia annua* simplified coding sequences (CDS) as reference using ASTRAL v. 5.6.3 (Mirarab et al., 2014). Local posterior probabilities are indicated near nodes. Branch lengths are in coalescent units. The colors of branches indicate the traditional subgeneric classification of Artemisia. Dashed line denotes differences to the topology obtained with SNP alignments allowing for more than 80% missing data at each marker after mapping to *Artemisia annua* genome (Figure 1).

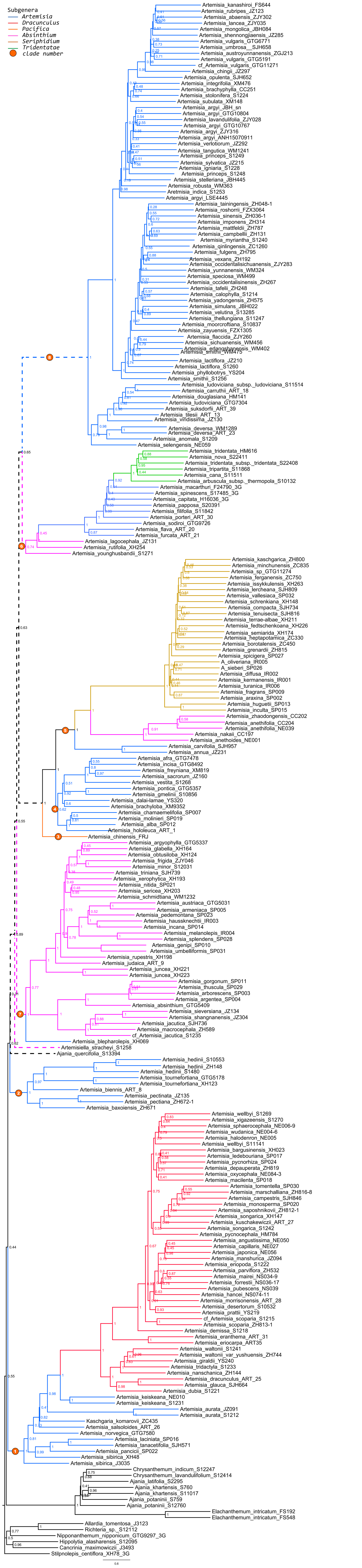

Fig. S11 Multigene coalescent species tree estimated from 540 nuclear gene trees based on single nuclear polymorphism (SNP) alignments (> 150 bp and containing ≥50% of the total number of samples) obtained with *Chrysanthemum seticuspe* simplified coding sequences (CDS) as reference using ASTRAL v. 5.6.3 (Mirarab et al., 2014). Local posterior probabilities are indicated near nodes. Branch lengths are in coalescent units. The colors of branches indicate the traditional subgeneric classification of *Artemisia*. Dashed line denotes differences to the topology obtained with SNP alignments allowing for more than 80% missing data at each marker after mapping to *Artemisia annua* genome (Figure 1).

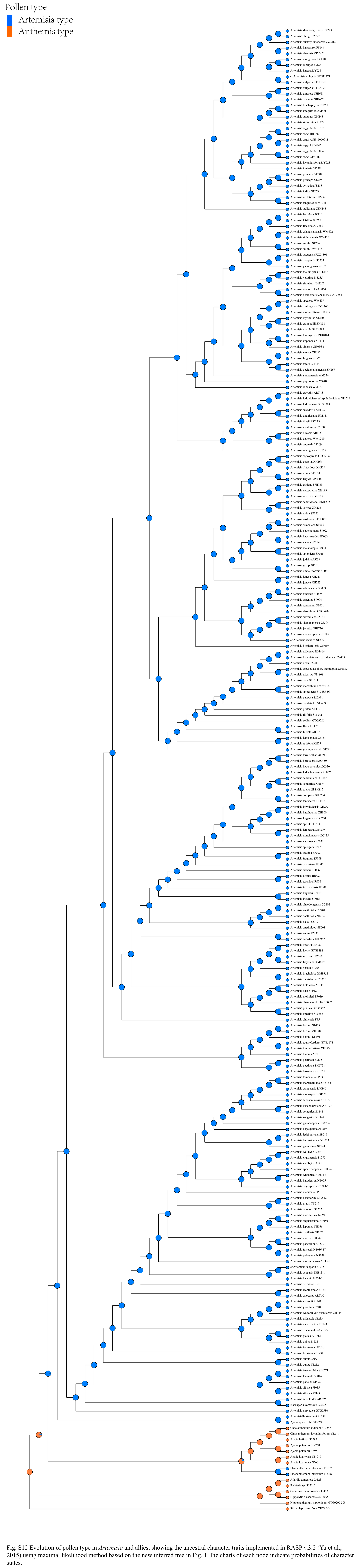

Fig. S12 Evolution of pollen type in *Artemisia* and allies, showing the ancestral character traits implemented in RASP v.3.2 (Yu et al., 2015) using maximal likelihood method based on the new inferred tree in Fig. 1. Pie charts of each node indicate probabilities of character states.

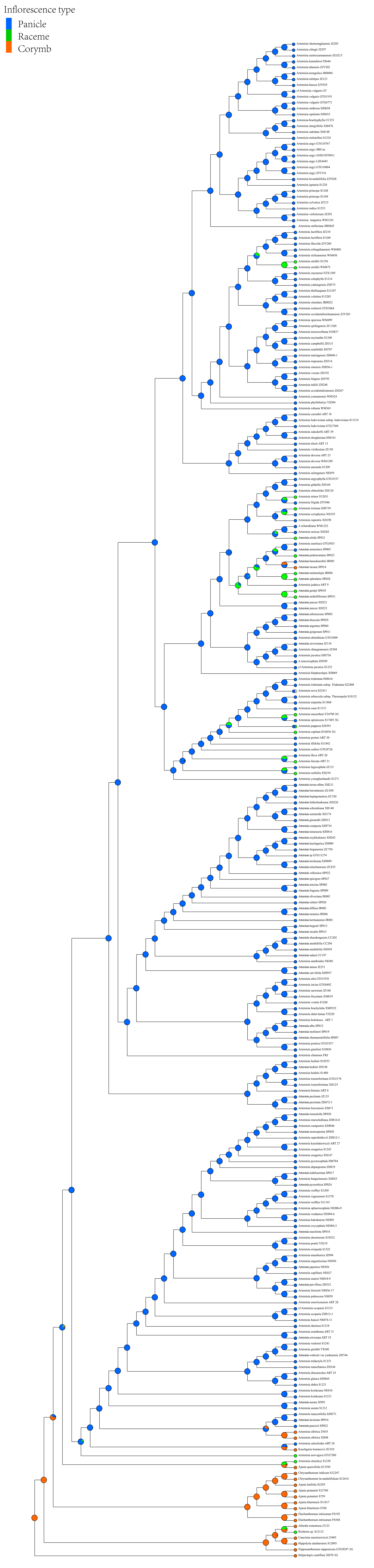

Fig. S13 Evolution of synflorescence type in *Artemisia* and allies, showing the ancestral character traits implemented in RASP v.3.2 (Yu et al., 2015) using maximal likelihood method based on the new inferred tree in Fig. 1. Pie charts of each node indicate probabilities of character states.

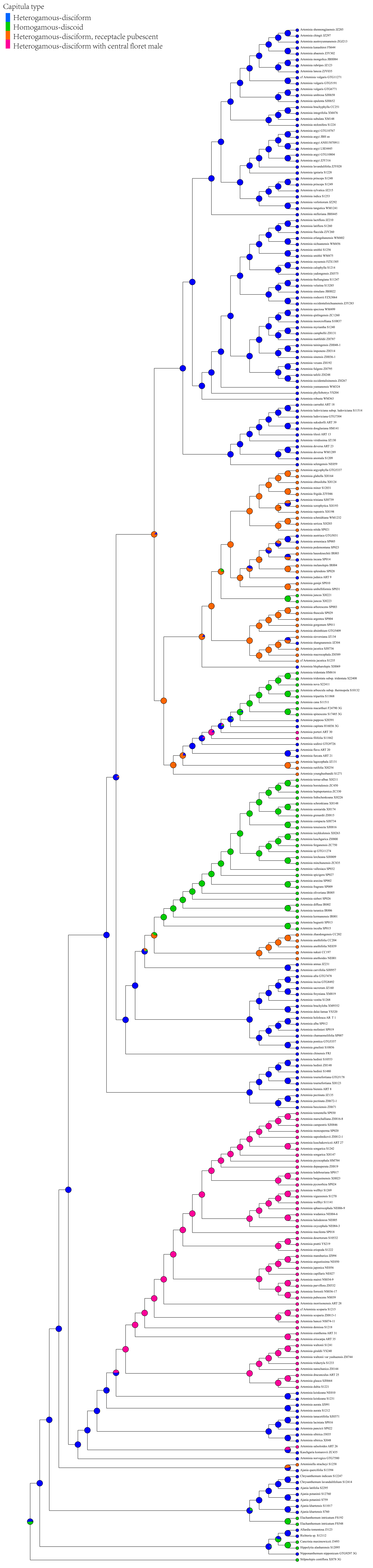

Fig. S14 Evolution of capitulum type in *Artemisia* and allies, showing the ancestral character traits implemented in RASP v.3.2 (Yu et al., 2015) using maximal likelihood method based on the new inferred tree in Fig. 1. Pie charts of each node indicate probabilities of character states.

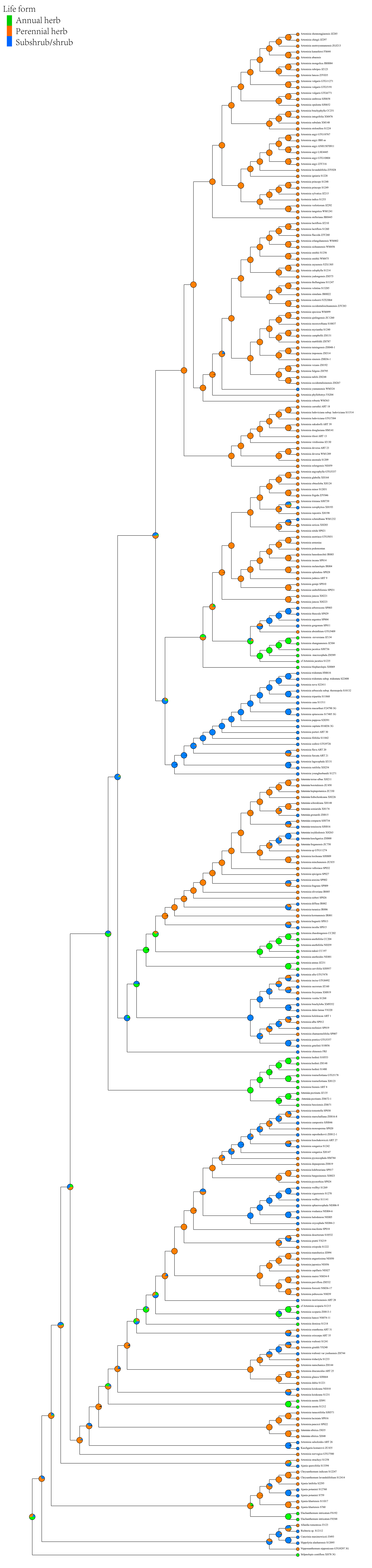

Fig. S15 Evolution of life form in *Artemisia* and allies, showing the ancestral character traits implemented in RASP v.3.2 (Yu et al., 2015) using maximal likelihood method based on the new inferred tree in Fig. 1. Pie charts of each node indicate probabilities of character states.

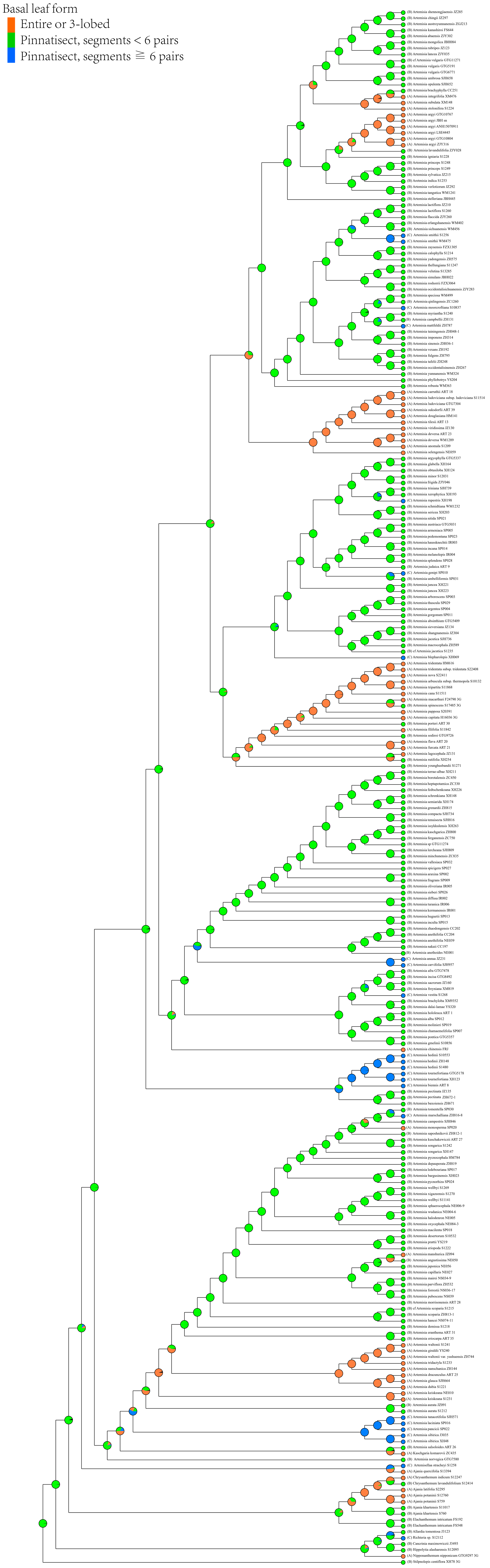

Fig. S16 Evolution of basal leaf form in *Artemisia* and allies, showing the ancestral character traits implemented in RASP v.3.2 (Yu et al., 2015) using maximal likelihood method based on the new inferred tree in Fig. 1. Pie charts of each node indicate probabilities of character states.

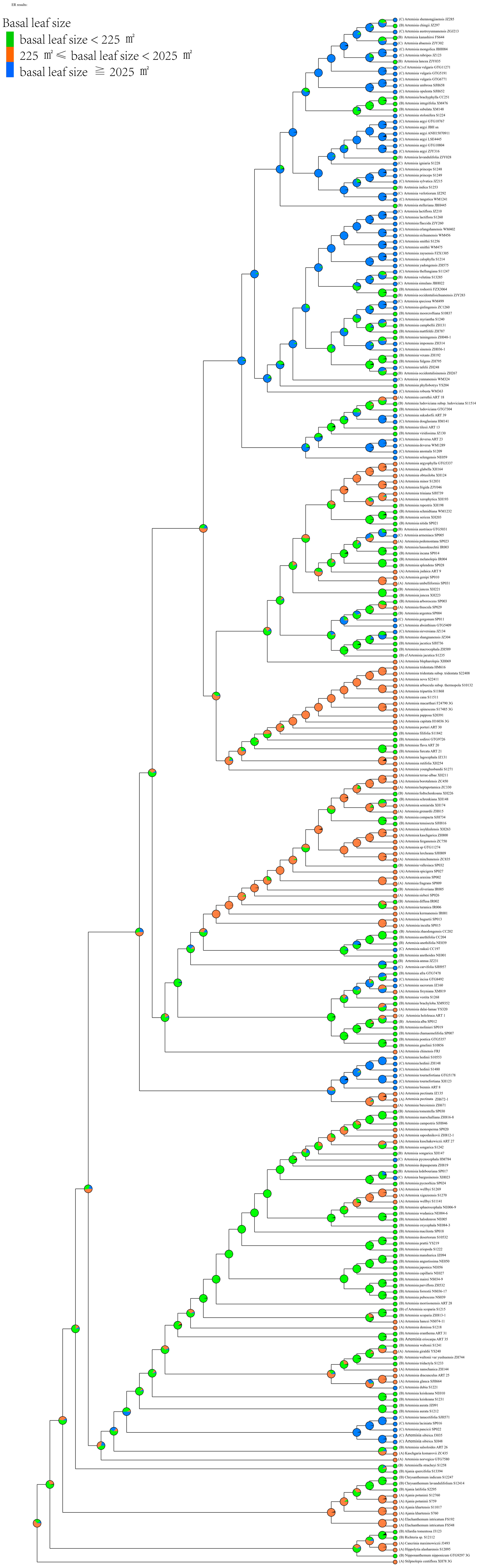

Fig. S17 Evolution of basal leaf size in *Artemisia* and allies, showing the ancestral character traits implemented in RASP v.3.2 (Yu et al., 2015) using maximal likelihood method based on the new inferred tree in Fig. 1. Pie charts of each node indicate probabilities of character states.
